# CEP250 is Required for Maintaining Centrosome Cohesion in the Germline and Fertility in Male Mice

**DOI:** 10.3389/fcell.2021.754054

**Published:** 2022-01-19

**Authors:** Sandrine Floriot, Laura Bellutti, Johan Castille, Pauline Moison, Sébastien Messiaen, Bruno Passet, Laurent Boulanger, Abdelhak Boukadiri, Sophie Tourpin, Christian Beauvallet, Marthe Vilotte, Julie Riviere, Christine Péchoux, Maud Bertaud, Jean-Luc Vilotte, Gabriel Livera

**Affiliations:** ^1^ INRAe, AgroParisTech, Université Paris-Saclay, GABI, Jouy-en-Josas, France; ^2^ Laboratory of Development of the Gonads, UMRE008 Genetic Stability Stem Cells and Radiations, IRCM/IBFJ CEA, Université de Paris, Université Paris-Saclay, Paris, France

**Keywords:** centrosome, spermatogenesis, meiosis, DNA damage, oogenesis

## Abstract

Male gametogenesis involves both mitotic divisions to amplify germ cell progenitors that gradually differentiate and meiotic divisions. Centrosomal regulation is essential for both types of divisions, with centrioles remaining tightly paired during the interphase. Here, we generated and characterized the phenotype of mutant mice devoid of *Cep250/C-Nap1*, a gene encoding for a docking protein for fibers linking centrioles, and characterized their phenotype. The *Cep250* ^-/-^ mice presented with no major defects, apart from male infertility due to a reduction in the spermatogonial pool and the meiotic blockade. Spermatogonial stem cells expressing Zbtb16 were not affected, whereas the differentiating spermatogonia were vastly lost. These cells displayed abnormal *γ*H2AX-staining, accompanied by an increase in the apoptotic rate. The few germ cells that survived at this stage, entered the meiotic prophase I and were arrested at a pachytene-like stage, likely due to synapsis defects and the unrepaired DNA double-strand breaks. In these cells, centrosomes split up precociously, with *γ*-tubulin foci being separated whereas these were closely associated in wild-type cells. Interestingly, this lack of cohesion was also observed in wild-type female meiocytes, likely explaining the normal fertility of *Cep250* ^-/-^ female mice. Taken together, this study proposes a specific requirement of centrosome cohesion in the male germline, with a crucial role of CEP250 in both differentiating spermatogonia and meiotic spermatocytes.

## Introduction

Gametogenesis displays striking sex-specific features in mammals (Teletin et al., 2017; Spiller and Bowles, 2019). Oogenesis is initiated during fetal life, with primordial germ cells rapidly initiating meiosis after a few rounds of mitotic divisions in the ovary. In males, spermatogenesis begins during postnatal life. Spermatogenesis is a cellular differentiation process that includes several coordinated steps that lead to the production of spermatozoa (Griswold, 2015). These steps comprise proliferation and differentiation of spermatogonia, spermatocyte meiosis, and post-meiotic differentiation of spermatids. The spermatogonial stem cells can self-renew and produce undifferentiated A-type spermatogonial progenitors that are committed to spermatogenesis. Undifferentiated A-type spermatogonia undergo a series of mitotic divisions, thereby amplifying the pool of undifferentiated A spermatogonia that can irreversibly transit to the differentiating spermatogonia. This transition is followed by five mitotic divisions, which lead to the formation of B-type differentiated spermatogonia, and a final mitotic division that results in the formation of preleptotene spermatocytes that further undergo meiosis. Meiosis is a succession of two cell divisions preceded by a single round of DNA replication, resulting in the exact halving of the chromosome number to produce haploid spermatids. This segregation of chromosomes requires homologous chromosome pairing and synapsis *via* a specialized form of homologous recombination during the meiotic prophase I (Baudat et al., 2013).

Centrosomes are cell structures consisting of two centrioles connected by fibrous linkers. They play a central role during cell division by acting as microtubule organizing centers (MTOCs) owing to the presence of *γ*-tubulin in the pericentriolar area (Azimzadeh and Bornens, 2007; Fu et al., 2015). CEP proteins play crucial roles in centriole biogenesis and cell cycle regulation. Mutations in human centrosomal proteins are most commonly associated with autosomal primary recessive microcephaly (MCPH) and Seckel syndrome. In somatic cells, centrosomes duplicate during the S phase, separate in the M phase, and then migrate to opposite poles from the mitotic spindle. In meiotic cells, centrosome duplication is delayed, occurring shortly after the pre-meiotic S phase (Alfaro et al., 2021; Wellard et al., 2021). Despite their importance, the function of centrosomes during gametogenesis has been poorly documented. The downregulation of CEP170 results in centrosome distribution defects in meiosis metaphase I and, consequently in aberrant spindle assembly (Qin et al., 2019). The deregulation of CEP55 expression results in a progressive loss of spermatogonial cells (Chang et al., 2010; Sinha et al., 2018; Hua et al., 2021).

The centrosome-associated protein CEP250, also known as C-Nap1, is a member of the CEP protein family (Mack et al., 1998). CEP250 is a key regulator of centrosome cohesion, which connects the two parent centrioles after their duplication (Bahe et al., 2005; Yang et al., 2006; He et al., 2013; Panic et al., 2015). This connection is established through a proteinaceous linker containing rootletin (CROCC) and LRRC45 filaments (Bahe et al., 2005; He et al., 2013; Vlijm et al., 2018). Recently, a truncating mutation of Cep250 in cattle has been associated with the Caprine-like Generalized Hypoplasia Syndrome, due to the loss of centrosome cohesion and centrosome splitting (Floriot et al., 2015). This syndrome associates with features similar to those of autosomal recessive primary microcephaly (MCPH) and Seckel-like syndrome. In humans, *CEP250* mutations are associated with Usher syndrome (Khateb et al., 2014; Fuster-García et al., 2018; Kubota et al., 2018).

In this study, we generated a null *Cep250* mouse strain to definitively characterise the mutant phenotype resulting from the loss of CEP250. Unexpectedly, we found that *Cep250* invalidation triggered male-specific infertility. CEP250 plays a crucial role during spermatogenesis in two different steps: in differentiating spermatogonia and during meiotic prophase I. This study highlights the relationship between centrosomal cohesion, mediated by CEP250, and the mitotic amplification of progenitors and meiotic prophase I progression specifically in the male germline.

## Materials and Methods

### Animals and Ethics Statement

All animal care procedures and experiments were carried out in accordance with the Guide for the Care and Use of Laboratory Animals (NIH Publication No.85-23, revised 1996) and were approved by the Local Animal Experimentation Ethics Committee and the French Ministry of Research (APAFIS#1215-2015090115013347 v5) and the Haut Conseil des Biotechnologies (HCB n°6461). Mice were housed under controlled photoperiod conditions (lights on from 08:00 to 20:00) and supplied with commercial food and tap water ad libitum. Embryos were collected from dated matings. Males were caged with females overnight, and the presence of a vaginal plug was examined the following morning. The following midday was defined as 0.5 day post conception (dpc). Mice were sacrificed *via* cervical dislocation, and fetuses were removed from the uterine horns before gonad isolation under a binocular microscope.

### TALEN Plasmid Constructs

*Cep250* invalidation was performed by using the transcription activator-like effector nuclease (TALEN) genome editing technology. The end of exon 5 of mouse *Cep250* gene was targeted at the site of the original mutation associated with Caprine-like Generalized Hypoplasia Syndrome (SHGC) phenotype in cattle (chromosome: GRCm38:2:155 958 456-155 998 900 (forward strand)). The target sequences were selected using the ZiFiT Targeter program (http://zifit.partners.org). A potential TALEN target sequence identified *via* this program was selected empirically, with a preference for an 18-16-18 combination (16 bases for the spacer). The chosen sequences were TAGTGAGGAAGGAGAGCC for the left TALEN and GGAGGTAGGAGAACAAGA for the right (localization 2:165538094-165538145). No homology with the entire targeted sequence was identified (BLAST NCBI) at any other location in the genome, which might represent a potential off-target site. The TALEN kit used for TALE assembly was a gift from the Keith Joung Laboratory (Addgene kit # 1000000017).

TALENs were constructed according to the restriction enzyme and ligation (REAL) assembly method, as described previously (Reyon et al., 2012). The left TALEN was constructed by assembling units of the kit in the following order: 6-14-20-24-26-14-19-21-26-14-19-21-29-11-19-22 (by groups of four units). The entire insert was subcloned into the final JDS71 plasmid, which was cleaved at the bsmb1 site. Similarly, the right TALEN was constructed by assembling units of the kit in the following order: 7-15-20-24-30-15-17-25-27-12-20-21-27-12-20-22. The entire insert was subcloned into the final JDS71 plasmid. All inserts of the final plasmids were sequenced with primers 2978 (TTG​AGG​CGC​TGC​TGA​CTG) and 2980 (TTA​ATT​CAA​TAT​ATT​CAT​GAG​GCA​C). To prepare RNA from each plasmid for microinjection, 5 μg of the TALEN plasmid was linearized with 20 U of Pme1 enzyme (New England Biolabs) for 8 h at 37°C in a 100 μL reaction volume. The linearized fragment was electrophoresed on an agarose gel and purified using the Qiagen Gel Extraction Column Kit (Qiagen, Hilden, Germany). Messenger RNA (mRNA) was prepared from 1 μg of purified linearized plasmid, using the ARCA T7 capRNA pol kit (Cellscript, TEBUbio, France), and polyadenylated with the polyA polymerase tailing kit (Epicentre) according to the manufacturer’s instructions. The synthesized mRNA was purified using the RNEasy mini kit (Qiagen) and resuspended in distilled water; then, mRNA concentration was estimated using a Nanodrop spectrophotometer (ND-1000, Thermo Scientific). The mRNA was then diluted to 100 ng/μL with injection buffer (Millipore, France) and stored at -80°C until further use.

### Generation of the CEP250 Transgenic Mouse Line Using TALENs

TALEN mRNAs (2 ng/µL) were microinjected into the pronuclei of fertilized eggs derived from superovulated C57BL/6 female mice crossed with males of the same strain. Embryos were transferred into the oviducts of pseudo-pregnant C57BL/6 female mice. The resulting offspring were genotyped by DNA analysis of tail biopsies, as described in the DNA sequencing subsection. Transgenic mice were crossed with C57BL/6 mice to derive F1 offspring, which were used to produce the *Cep250* transgenic line.

### DNA Isolation

The tail tips of mice were lysed in 100 mM Tris-HCl (pH 7.5), 5 mM EDTA, 0.2% SDS, and 200 mg/ml proteinase K overnight at 37°C and centrifuged for 10 min at 13,000 g. Genomic DNA was precipitated with an equal volume of isopropanol and resuspended in 10 mM Tris-HCl (pH 7.5) and 0.1 mM EDTA. DNA concentration was measured using a NanoDrop ND-1000 spectrophotometer.

### DNA Sequencing of Founder Newborns

Primers were designed, using Primer3 software, in a manner so that they annealed ∼150 bp upstream and downstream from the expected TALEN-target site. PCR was carried out in a final volume of 60 µL using 200 ng of DNA and primers, which amplified a 333 pb fragment of *Cep250* surrounding the target site in exon 5 (for primer sequences, Supplementary Table S1), with standard GoTaq PCR reagents (Promega) on a Master Thermal Cycler (Eppendorf). The cycling conditions were as follows: initial denaturation at 94°C for 2 min, followed by 35 cycles at 94°C for 20 s, 58°C for 30 s, 72°C for 30 s. Sanger sequencing was performed using standard methods (Eurofins) to identify null mutations.

### Mouse Genotyping

Transgenic mice were genotyped using the same pair of primers against *Cep250* surrounding the TALEN restriction site and the same PCR protocol, as described above. The PCR products were precipitated and then digested with the FokI restriction enzyme (New England Biolabs). This enzyme recognizes the GGATG DNA sequences and thus only cleaves wild-type PCR products into two fragments. PCR products were electrophoresed on a 2% agarose gel.

### Tissue Collection

At 5–21 weeks of age, the wild-type and Cep250^-/-^ mice were euthanized, and various tissues were dissected, weighed, and either frozen at −80°C for RNA/protein extraction, or rinsed in phosphate-buffered saline (PBS), fixed overnight in 4% paraformaldehyde (PFA)/PBS, and embedded in paraffin (EG 1160 Embedding Center; Leica).

### Semi-Quantitative Reverse Transcription–PCR (RT–PCR)

Total RNA was extracted from the kidneys of wild*-*type and transgenic animals using the RNA NOW reagent (Ozyme). After DNase I treatment, the RNA (200 ng) was reverse transcribed into complementary DNA (cDNA) using the Superscript II kit (Invitrogen) with oligo (dT) according to the manufacturer’s instructions. The cDNA was then amplified *via* PCR with primers for the *Cep250* and *Gapdh* genes (Supplementary Table S1). Primers were designed, using Primer3 software (http://bioinfo.ut.ee/primer3-0.4.0/primer3/), from separate exons to produce 100–300 bp amplicons. The *Gapdh* gene was used as a reference to normalize the relative expression of the target genes.

### Quantitative RT–PCR (RT–qPCR)

Fetal and postnatal gonads were harvested and frozen in RLT lysis buffer for RNA extraction. Total RNA was extracted using the RNeasy Mini Kit (QIAGEN, Valencia, CA, United States). cDNA was obtained *via* reverse transcription using the High-Capacity cDNA Reverse Transcription Kit (Applied Biosystems, Foster City, CA, United States) according to the manufacturer’s instructions. The 7900HT fast real-time PCR system (Applied Biosystems) and SYBR Green chemistry were used for RT–qPCR. The comparative Ct method was used to determine the relative quantities of mRNA, using *β-actin* or *DEAD-Box Helicase 4* (*Ddx4*) mRNA as the endogenous reporter. The results are presented as a percentage of the control (i.e., expression of the control is defined as 1). Each RNA sample was analyzed in duplicate. All primers were used at a final concentration of 400 nM. The sequences of oligonucleotides used in this study are listed in Supplementary Table S1.

### Western Blot Analysis

To isolate the centrosomal fraction from the testis, we followed the protocol described by Moudjou and Bornens (Moudjou and Bornens, 1998). Briefly, we crushed the testes using ultra-turrax in 2.5 ml of a solution containing 10 mM Tris-HCl (pH 7.2), 2 mM MgCl_2_, 0.5% NP-40, and 1 mM DTT with EDTA-free Halt Protease Inhibitor Cocktail (Thermo), at 4°C. All the subsequent steps were performed at 4°C. Chromatin and nuclei were pelleted *via* centrifugation at 2,500 × g for 10 min. The supernatant was deposited on 5 ml of a 50% sucrose cushion (w/w) dissolved in 10 mM Tris-HCl, pH (7.2), 0.1% Triton X-100, and 1 mM DTT. The centrosomes were concentrated onto the sucrose cushion *via* centrifugation at 22,500 × g for 20 min. The supernatant was removed, and the sucrose cushions were pooled, including the interface containing the centrosomes. We then transferred the solution onto a discontinuous sucrose gradient comprising 10 ml of 70% sucrose (w/w), 6 ml of 40% sucrose (w/w), and 6 ml of 30% sucrose (w/w), in 10 mM Tris-HCl (pH 7.2) and 1 mM DTT, and centrifuged it at 116,000 × g for 75 min. Aliquots were collected from each fraction, in Eppendorf tubes, for analysis to identify the centrosome-containing fractions. The 2-D Quant kit (GE Healthcare) was used to determine protein concentration. Protein samples (50–100 µg) were mixed with 2× Laemmli and separated on a 7.5% polyacrylamide home-cast SDS-PAGE at 80 V for 20 min and then at 150 V until the front reached the bottom of the gel; a pink pre-stained protein ladder (Nippon Genetics) was used a reference. The resolved proteins were transferred onto a nitrocellulose membrane using Trans-Blot Turbo (Bio-Rad) at 2.5 A and 25 V maximum for 10 min. The proteins were then stained with a 5× Red Ponceau solution and destained in TBS-Tween 20 and 0.1% (TBST) for 15 min. Next, the membrane was blocked with 5% non-fat lyophilized milk in TBST for 1 h at room temperature, following which primary hybridization was carried out with the monoclonal antibody anti-CEP250 C-Nap1 (Santa Cruz; Sc390540), dissolved in TBST, for 1 h at room temperature. After three washes in TBST, the membrane was incubated with a horseradish peroxidase-conjugated secondary antibody. Signals were detected using the ECL prime enhanced chemiluminescence system (GE Healthcare) and a ChemiDoc Touch Imager system (Bio-Rad).

### Histology, Immunohistochemistry, and Immunofluorescence

For histological analysis, the gonads were fixed in 4% formaldehyde. The fixed gonads were dehydrated, embedded in paraffin, and cut into 5 μm thick sections. The sections were mounted on slides, dewaxed, and rehydrated, following which they were boiled for 20 min in citrate buffer.

For immunohistochemical staining, the endogenous peroxidase activity was blocked by incubating the sections for 15 min with 3% hydrogen peroxide. The sections were then blocked with 2% horse serum for 30 min and incubated with primary antibodies overnight at 4°C (Supplementary Table S2 for antibodies). After three washes with PBS, the slides were incubated with peroxidase-conjugated secondary antibodies (ImmPRESSTM reagent kit, Vector Laboratories) for 30 min at room temperature. Antibodies were detected using DAB (DAB substrate reagent kit, Vector Laboratories) or the VIP Vector (VIP substrate reagent kit, Vector Laboratories). All counts were carried out blind and were done using Histolab analysis software (Microvision Instruments, Evry, France).

For immunofluorescence staining, sections were blocked for 1 h in a gelatin-blocking solution, containing 0.2% BSA, 0.2% gelatin, and 0.05% Tween in 1× PBS, and incubated with an appropriate primary antibody, diluted in the blocking solution, for 1.5 h at 37°C. After three washes with PBS containing 0.05% Tween, the slides were incubated with secondary antibodies, diluted in the blocking solution, at 37°C for 1 h. The slides were then incubated with DAPI and mounted in the ProLong Gold antifade mounting medium (Thermo Fisher). Imaging was performed using a Leica DM5500 B epifluorescence microscope (Leica Microsystems) equipped with a CoolSNAP HQ2 camera (Photometrics) and Leica MMAF software (Metamorph) or a confocal microscope (Spinning disc). Images were processed using the ImageJ software.

Pericentrin immunostaining was performed with dissociated testicular cells. After removal of tunica albuginea, testes were incubated in 1 mg/ml PBS-collagenase solution for 20 min at 32°C with pipeting up and down regularly. Dissociated cells were washed twice in PBS. Cells were counted using Malassez chamber with Trypan staining and placed in Cytospin device to reach 25,000 cell per slide. Samples were then fixed with 4% PFA solution. Cells were permeabilized by a 5 min incubation in 0.2% Triton X-100 solution and washed in PBS. Subsequent steps were performed as described above.

### Preparation of the Spermatocyte Chromosome Spreads

The testes used for spermatocyte spread preparation were collected from postnatal mice at 12 days post-partum (dpp). Cells were isolated *via* enzymatic digestion. Briefly, the albuginea was removed; then, the seminiferous tubules were isolated and incubated at 32°C for 1 h in the TrypLE Express reagent (Thermo Fisher) by pipetting regularly. Fetal bovine serum was used to terminate the TrypLe reaction. The isolated cells were resuspended in 0.1 M sucrose. The sucrose suspension was distributed on slides in humid chambers and fixed with 1% PFA (in H_2_O, pH 9.2) and 0.001% Triton X-100 for 1.5 h. After fixation, the slides were rinsed with H_2_O and 0.4% Photo-Flo 200 (Kodak).

After washing the spermatocyte chromosome spreads, the slides were air-dried and incubated with a gelatin-blocking solution (containing 0.2% BSA, 0.2% gelatin, and 0.05% Tween in 1× PBS) for 1 h at room temperature and then with primary antibodies, dissolved in blocking solution, overnight at room temperature (Supplementary Table S2 for the list of antibodies). Next, the slides were incubated with secondary antibodies at 37°C for 1.5 h, stained with DAPI, and mounted on the Prolongold medium. Imaging was performed using a Leica DM5500 B epifluorescence microscope (Leica Microsystems) equipped with a CoolSNAP HQ2camera (Photometrics) and Leica MMAF software (Metamorph). Images were processed using the ImageJ software.

## Results

### CEP250 is Expressed in Male Germ Cells

**FIGURE 1:**
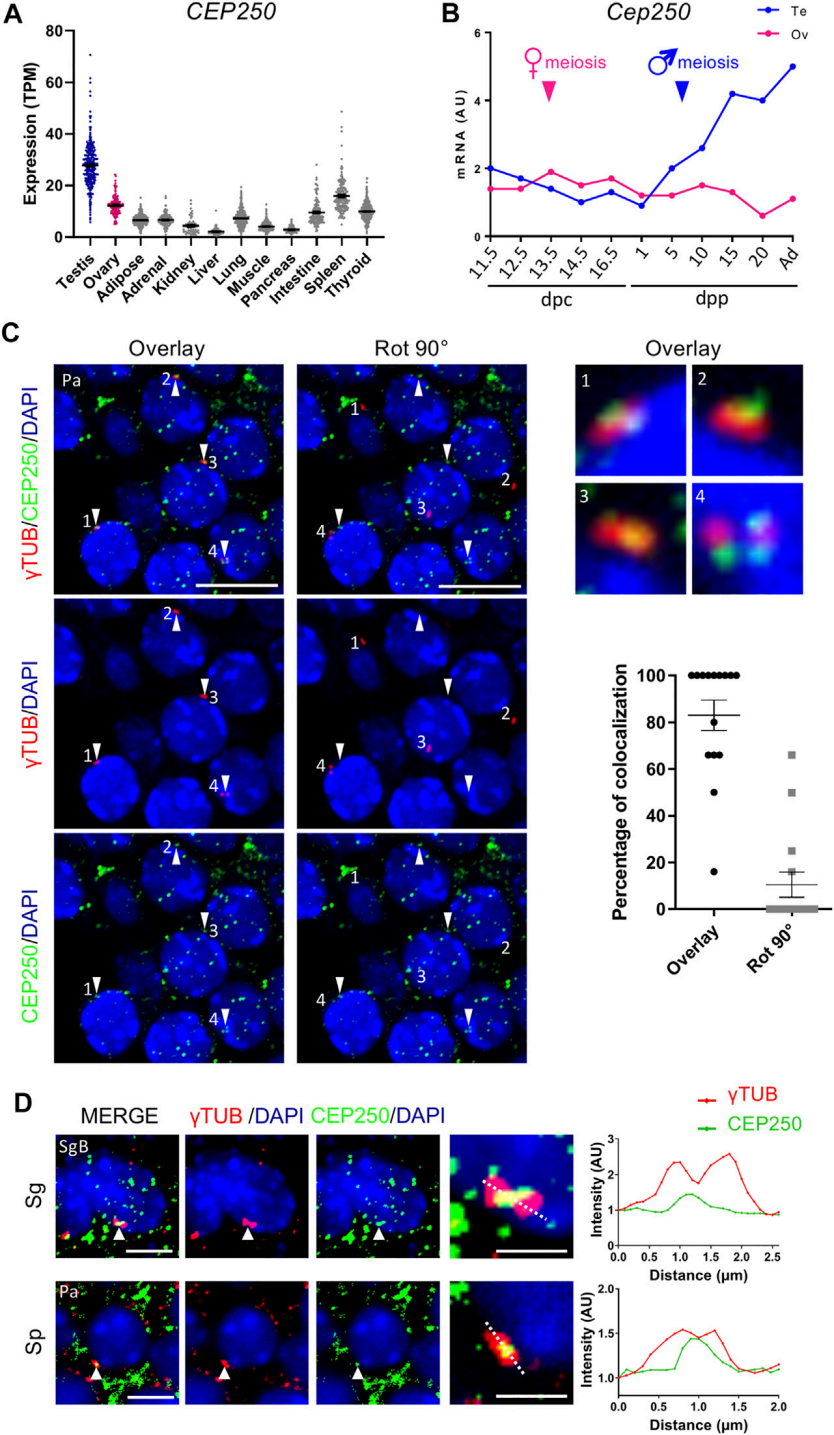
Cep250 is expressed in male germ cells. **(A)** Expression (Transcripts Per Million, TPM) of *Cep250* in different human tissues. **(B)** *Cep250* mRNA expression pattern in mouse gonads. mRNA expression was mesured by RT-qPCR and normalized to *β-actin* expression in whole fetal and postnatal testes (Te, blue) and ovaries (Ov, magenta) at the indicated developmental ages (days post coitum, dpc; days post-partum, dpp). Values are expressed in Arbitraty Units (AU). **(C)** Immunofluorescence staining of *γ*-tubulin (*γ*TUB, red) and CEP250 (green) in the 15 dpp testis sections. DNA is stained with DAPI. 90° clockwise rotation (Rot 90°) of *γ*-tubulin. White arrowheads indicate CEP250. Numbers indicate *γ*-tubulin. Magnification of the *γ*-tubulin and CEP250 stained centrosomes in right images. Right graph quantification of the *γ*-tubulin and CEP250 overlay. Each point or square corresponds to the percentage of colocalization per image. Pa: pachytene spermatocytes. Scale bar: 5 µm. **(D)** Immunofluorescence staining of *γ*-tubulin (*γ*TUB, red) and CEP250 (green) in the spermatogonia (Sg) and spermatocytes (Sp) from 15 dpp testis. DNA is stained with DAPI. Scale bar: 5 µm. Magnification of *γ*-tubulin-stained centrosomes in the right panel. Scale bar: 2 µm. A dotted line was traced through *γ*-tubulin-stained centrosomes, and intensities of *γ*-tubulin (red) and CEP250 (green) were quantified using the ImageJ software (Plot profile). Values are expressed in Arbitraty Units (AU). SgB, B spermatogonia.

In order to assess Cep250 abundance in various tissues, we first analyzed the publicly available RNA-seq datasets for human adult tissues. The testis displayed the highest *Cep250* expression compared to the somatic tissues (Figure 1A). To refine *Cep250* expression profile in the gonads, *Cep250* expression was quantified *via* RT–qPCR at various stages in mouse fetal and postnatal gonads. *Cep250* expression levels increased in post-natal testes from 5 dpp and onwards, indicating the possible roles of CEP250 in spermatogenesis and meiosis (Figure 1B). Next, we investigated the localization of CEP250 protein in post-natal testis sections. Consistent with its known centrosomal role, CEP250 colocalized with *γ*-tubulin in the spermatogonia and spermatocytes (Figures 1C,D; Supplementary Figure S1). Upon rotating the *γ*-tubulin image 90° clockwise relative to the CEP250 image, the existence of a specific signal for CEP250 at the centrosome was confirmed. CEP250 was localized between the two *γ*-tubulin foci which supported its possible role in centrosome cohesion during spermatogenesis (Figure 1D). Owing to the poor specificity of the signal, we could not exclude the possibility of the presence of CEP250 in other cellular compartments.

### CEP250 is Required for Spermatogenesis

**FIGURE 2:**
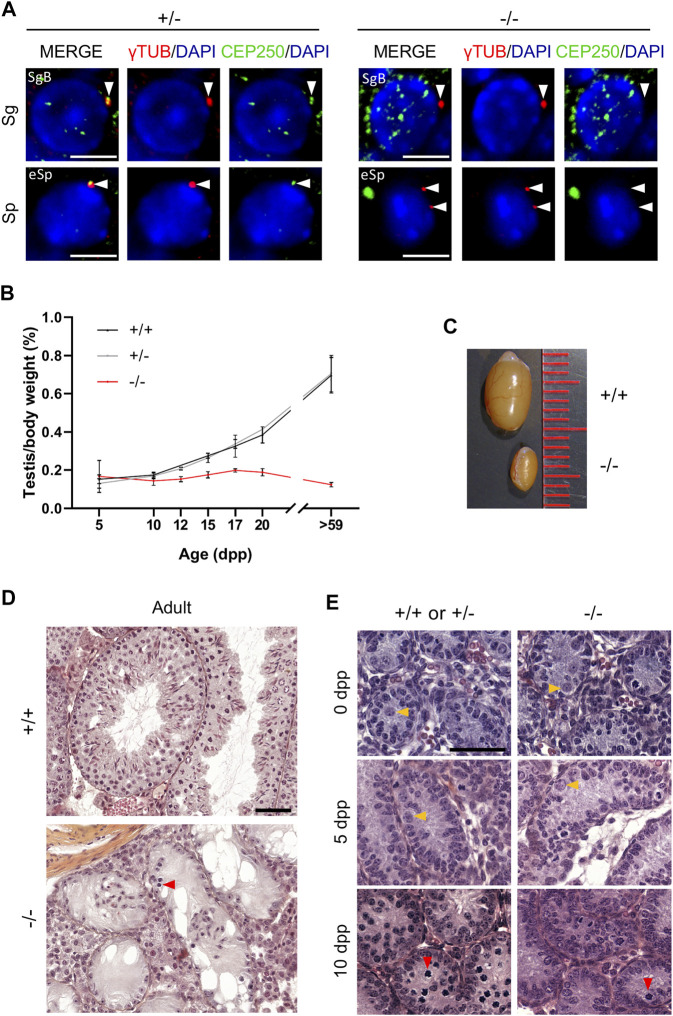
Cep250 is required for post-natal spermatogenesis. **(A)** Immunofluorescence staining of *γ*-tubulin (*γ*TUB, red) and CEP250 (green) in the control (+/-) and mutant (-/-) testis sections. DNA is stained with DAPI. White arrowheads indicate *γ*-tubulin stained centrosomes. Note that no CEP250 staining is observed in centrosomes from mutant spermatogonia (Sg) or spermatocytes (Sp). SgB, B spermatogonia; eSp, early spermatocytes (leptonema to early pachynema). Scale bar: 5 µm. **(B)** Analysis of testis weight/body weight from 5 to >59 day-old wild-type (+/+), heterozygous (+/−) and homozygous mutant (−/−) mice. Values correspond to mean ± SD, *n* = 3 to 25, except for +/− at 12, 20 and >59 dpp and −/− at 17 dpp (*n* = 2). **(C)** Adult testes from mutant (−/−) mice are smaller than the control (+/+) adult testes. **(D)** Hematoxylin/eosin-stained sections of the control (+/+) and mutant (−/−) adult testes. The red arrowhead indicates a spermatocyte in the mutant testis. Scale bar: 50 µm. **(E)** Hematoxylin/eosin-stained sections of the control (+/+ or +/−) and mutant (−/−) testes at the indicated developmental ages (0, 5 and 10 days post-partum, dpp). 0 dpp control is +/+; 5 dpp control is +/−; 10 dpp control is +/+. Yellow arrowheads indicate spermatogonia; red arrowheads indicate spermatocytes. Scale bar: 50 µm.

To further investigate the biological function of CEP250, we generated *Cep250* mutant mice in a C57BL/6 background. *Cep250* invalidation was performed by targeting exon 5 using the TALEN genome editing technology (Supplementary Figure S2A). The *Cep250* mutant founder allele showed a 6-bp deletion and a 1-bp insertion, which resulted in the disruption of the open reading frame, thus inducing a premature stop codon (Supplementary Figures S2B,C). The heterozygous animals were healthy and were used to generate homozygous mice. Homozygou*s* mutant mice developed normally, were viable, and were obtained at the expected Mendelian ratio. RT-PCR analysis revealed a considerable reduction in *Cep250* expression (testis and kidney), while western blotting revealed a complete absence of CEP250 (testis) in homozygous mice (Supplementary Figures S3A–C). We confirmed the absence of CEP250 protein in mutant testes *via* the immunofluorescence staining of seminiferous tubule sections (Figure 2A). Thus, these animals could be considered as null mutants (*Cep250* ^*-/-*^)*. Cep250* ^*-/-*^ male mice were fully infertile (Supplementary Table S3). Mutant testes were smaller than wild-type testes starting from 12 dpp (Figure 2B; Supplementary Figure S3D). The size difference was maximal during adulthood, with mutant testes being approximately 80% smaller than wild-type testes (Figures 2B,C; Supplementary Figure S3E). Notably, no difference was observed between the wild-type and *Cep250* heterozygous testes. Thus, we used heterozygous mice as controls in subsequent experiments. The histological analysis of adult *Cep250* ^*-/-*^ testis sections revealed complete absence of spermatids and spermatozoa in the seminiferous tubules (Figure 2D). Spermatocytes were exceedingly rare in mutant testes, and the few observed were arrested early during the meiotic prophase I (Supplementary Figure S4A). To better define the ontogenesis of testicular defects, postnatal testes were analyzed at 0, 5, and 10 dpp. At 0 and 5 dpp, no defects were observed in mutant testes (Figure 2E). At 10 dpp, when the first germ cells reached the early stages of meiotic prophase I, the density of leptotene and zygotene cells was substantially reduced in mutant testes (Figure 2E). These findings suggest that *Cep250* invalidation impairs spermatogenesis, both by reducing the number of germ cells reaching meiosis and preventing the completion of meiotic divisions.

### Germ Cell Loss in Cep250^-/-^ Mice

**FIGURE 3:**
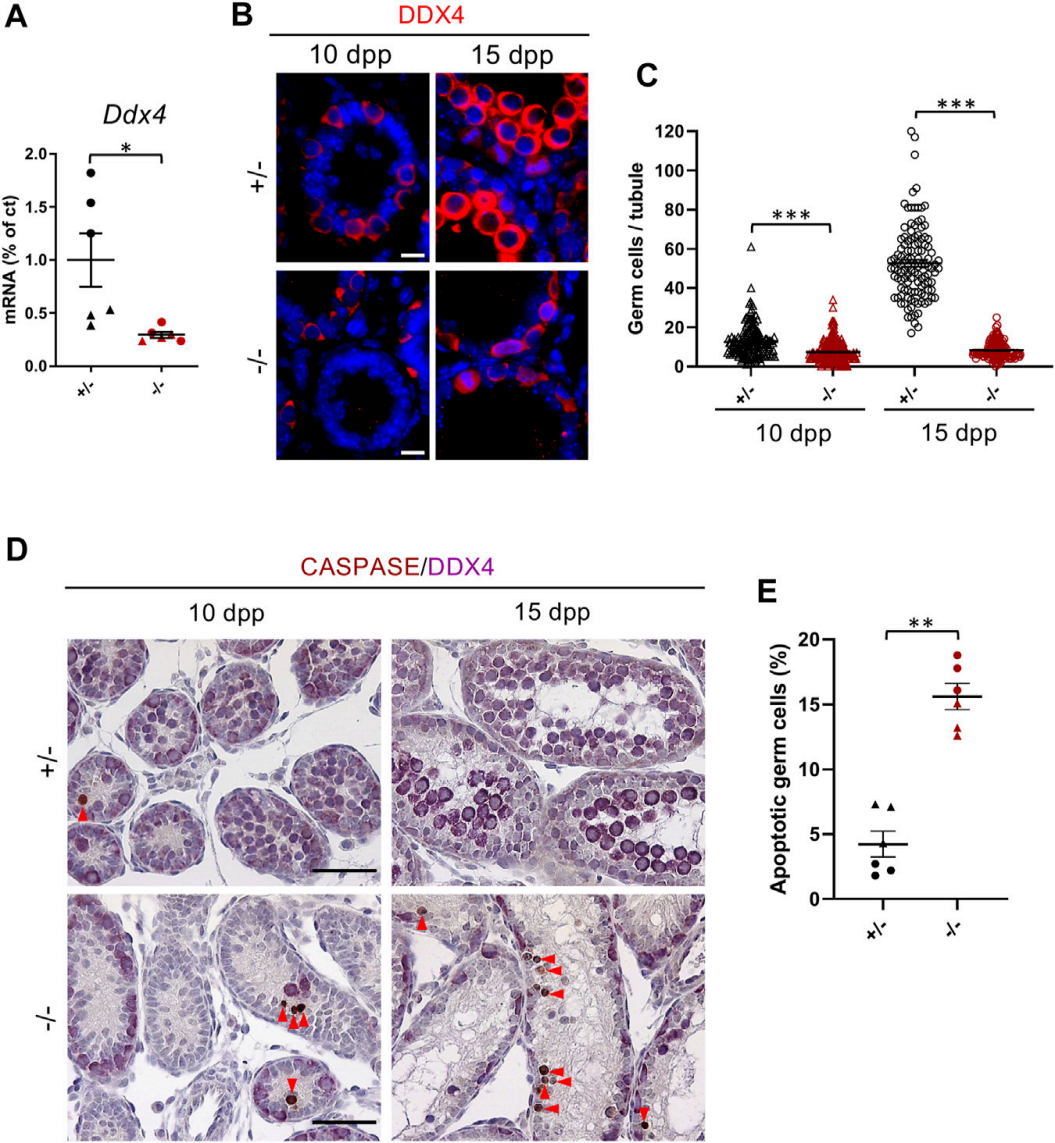
Germ cell number is decreased in *Cep250* ^*-/-*^ testes. **(A)** *Ddx4* mRNA expression levels were measured by RT-qPCR in the post-natal testes. Triangles represent 10 dpp values and circles represent 15 dpp values. *β-actin* was used as an endogenous reporter. Data are expressed as a percentage of the control expression level. Mean ± SEM. ***p < 0.001 (two-way ANOVA). **(B)** Immunofluorescence analysis of DDX4 (red) in 10 dpp and 15 dpp testis sections. DNA is stained with DAPI. Scale bar: 10 µm. **(C)** Quantification of the number of germ cells par tubule in 10 dpp and 15 dpp testis sections. Mean ± SEM. ***p < 0.001 (two-way ANOVA). **(D)** Histological analysis of 10 dpp and 15 dpp testis sections stained for DDX4 (purple) and the apoptotic marker cleaved caspase 3 (brown). Red arrowheads denote the germ cells stained for cleaved caspase 3. Scale bar: 50 µm. **(E)** Quantification of the percentage of apoptotic germ cells. Triangles represent 10 dpp values and circles represent 15 dpp values. Mean ± SEM. **p < 0.01 (two-way ANOVA).

To determine the spermatogenic stages that were altered in *Cep250* ^*-/-*^mice, we analyzed DDX4 (VASA) expression at 10 and 15 dpp, when germ cells were exclusively spermatogonia or early spermatocytes. DDX4 is used as a germ cell marker, as this RNA helicase is found in the germline of all animals, and in embryonic germ cells, up to the level of gametes, in mice. *Ddx4* mRNA levels were significantly reduced by 42 and 79%, respectively, in the 10 and 15 dpp mutant testes compared to those in heterozygous testes (Figure 3A). DDX4 immunostaining revealed fewer positive cells in both 10 and 15 dpp mutant testes (Figures 3B,C). In addition, many apoptotic cells that stained for cleaved caspase 3 were detected in the tubules of *Cep250* ^*-/-*^ testes (Figures 3D,E), explaining the reduction in germ cell population detected in the mutant testes. Germ cell apoptosis was also observed in the adult *Cep250* ^-/-^ testis (Supplementary Figure S4B). As germ cell survival is partly dependent upon the somatic niche, somatic cells in the mutant testes were analyzed using the Sertoli cell marker GATA1 and the common Sertoli and Leydig cell marker GATA4. Both GATA1 and GATA4 were unaffected in the post-natal mutant testes (Supplementary Figure S5A). Similarly, mRNA levels of the somatic cell-specific transcripts *Sox9* (Sertoli cells), *Gata4*, *Hsd3β* (Leydig cells), and *αSMA* (peritubular myoid cells) were unaltered in the mutant compared to that in the control testes (Supplementary Figure S5B).

Altogether, these results indicate that CEP250 is essential for post-natal germ cell survival and development without affecting somatic cell development, suggesting an intrinsic role of *Cep250* in germ cells.

### Cep250 is Essential for Late Differentiation of Spermatogonia

**FIGURE 4:**
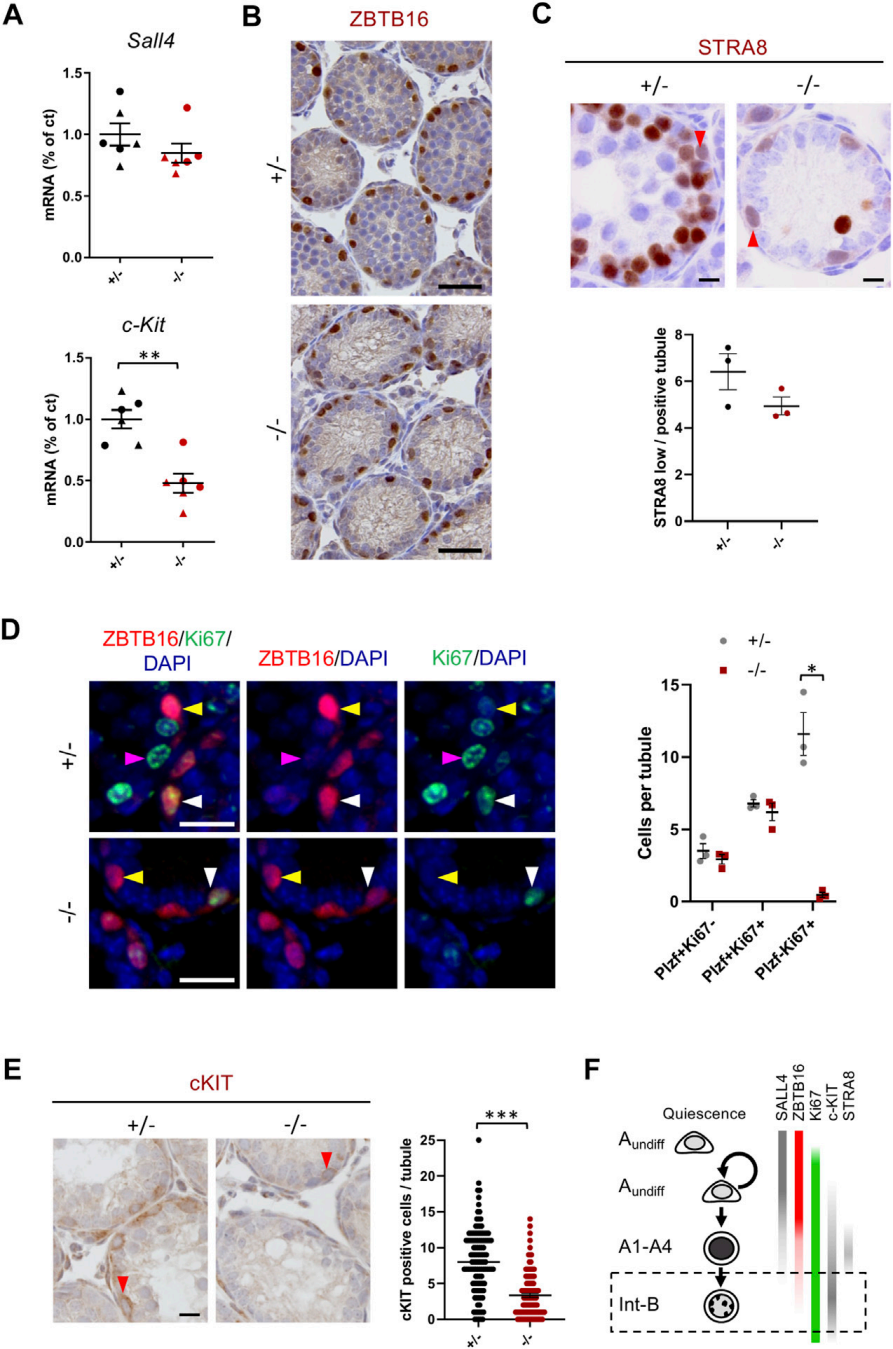
The differentiated spermatogonia are altered in *Cep250* ^*-/-*^. **(A)** mRNA expression levels of the undifferentiated (*Sall4*) and differentiated (*c-Kit*) spermatogonia cell markers were measured by RT-qPCR in the post-natal testes. Triangles represent 10 dpp values and circles represent 15 dpp values. *β-actin* was used as an endogenous reporter. Data are expressed as a percentage of the control expression level. Mean ± SEM. **p < 0.01 (two-way ANOVA). **(B)** Histological analysis of 15 dpp testis sections stained for the undifferentiated and A-type differentiating spermatogonial cell marker ZBTB16 (brown). Scale bar: 50 µm. **(C)** Histological analysis of 15 dpp testis sections stained for the cell marker STRA8 (brown), expressed at low level in the differentiating A-type spermatogonia (red arrowheads) and at high level in the preleptotene cells. Scale bar: 5 µm. Quantification of the number of STRA8 low-positive cells per positive tubule is shown in the graph. **(D)** Immunofluorescence analysis of the spermatogonial cell markers ZBTB16 (red) and Ki67 (green) in 15 dpp testis sections. DNA is stained with DAPI. Yellow arrowheads indicate spermatogonia stained exclusively for ZBTB16 (ZBTB16+Ki67-); white arrowheads indicate spermatogonia stained for both ZBTB16 and Ki67 (ZBTB16+Ki67+); magenta arrowheads indicate germ cells stained exclusively for Ki67 (ZBTB16-Ki67+). Quantification is shown in the right panel. Mean ± SEM. *p < 0.05 (two-way ANOVA). Scale bar: 20 µm. **(E)** Histological analysis of 15 dpp testis sections stained for differentiating spermatogonia cell marker cKIT (brown). Red arrowheads indicate spermatogonia stained for cKIT. Scale bar 5 µm. Quantification of the number of cKIT positive cells per tubule is shown in the right panel. Mean ± SEM. **p < 0.01 (two-way ANOVA). **(F)** Schematic representation of different markers expressed during spermatogonia differentiation. The dashed rectangle represents the population of germ cells impaired in the *Cep250* ^*-/-*^ male mice.

To further characterize the stages of spermatogenesis affected by *Cep250* invalidation, we performed RT-qPCR and immunostaining of different spermatogonia cell markers. mRNA levels of the undifferentiated spermatogonial marker *Sall4* were unaltered, while *c-Kit*, expressed in differentiated spermatogonia, was significantly downregulated in the 10 and 15 dpp testes (Figure 4A). The population of undifferentiated and differentiating type A spermatogonia, stained for ZBTB16, was equivalent in the control and mutant postnatal testes (Figure 4B). STRA8 is a marker found both at low levels in differentiating type A spermatogonia, and at high levels in pre-leptotene cells (Zhou et al., 2008; Endo et al., 2015). As expected, STRA8 was expressed in type A spermatogonia and strongly expressed in preleptotene spermatocytes in the mutants. Quantification of STRA8-low spermatogonia cells revealed no significant difference in Cep250^-/-^ testes (Figure 4C). Spermatogonia can be divided into three different populations according to Ki67 and ZBTB16 staining. The Ki67-negative and ZBTB16-positive cells correspond to quiescent or slow-proliferating spermatogonial stem cells. The Ki67- and ZBTB16-positive cells correspond to the proliferating, undifferentiated spermatogonia or the differentiating type A spermatogonia, in which ZBTB16 staining progressively declines. The Ki67-positive and ZBTB16-negative cells represent a more differentiated population of spermatogonia. Interestingly, we observed a 24-fold reduction in the population of Ki67-positive and ZBTB16-negative differentiating spermatogonia in the mutants (Figure 4D; Supplementary Figure S6A), with no alteration in the number of the earlier spermatogonium populations. Additionally, in the control testes, we observed clusters of ZBTB16-negative pH3-stained mitotic metaphase cells, corresponding to dividing late-differentiating spermatogonia (Supplementary Figure S7). However, this was hardly observed in the *Cep250* ^*-/-*^ testes, in which the pH3-positive spermatogonia were always ZBTB16-positive. Lastly, immunohistochemical detection of c-KIT confirmed the loss of differentiated spermatogonia (Figure 4E). Altogether, these findings indicate that the first spermatogonial divisions usually take place in the mutant testes, and that the differentiated spermatogonia are mostly altered (Figure 4F).

Surprisingly, we found that the differentiating spermatogonia with faint ZBTB16 staining were frequently positive for *γ*H2AX, a DNA double-strand break (DSB) marker, specifically in the mutant testes (Supplementary Figure S8A). Most ZBTB16-positive metaphases were also *γ*H2AX-positive in the mutants (Supplementary Figures S8A,B). This supports the hypothesis that spermatogonia undergo abnormal mitotic divisions, which could result in genome instability and abnormal *γ*H2AX in late spermatogonia. Overall, these results allowed us to identify the stages of spermatogonia divisions affected by *Cep250* invalidation. We found that a reduction in the population of late differentiating spermatogonia and the defects in dividing spermatogonia, likely induced the death of most germ cells before completing spermatogonia differentiation.

### The Mitosis to Meiosis Switch is Not Altered in Cep250^-/-^ Germ Cells

**FIGURE 5:**
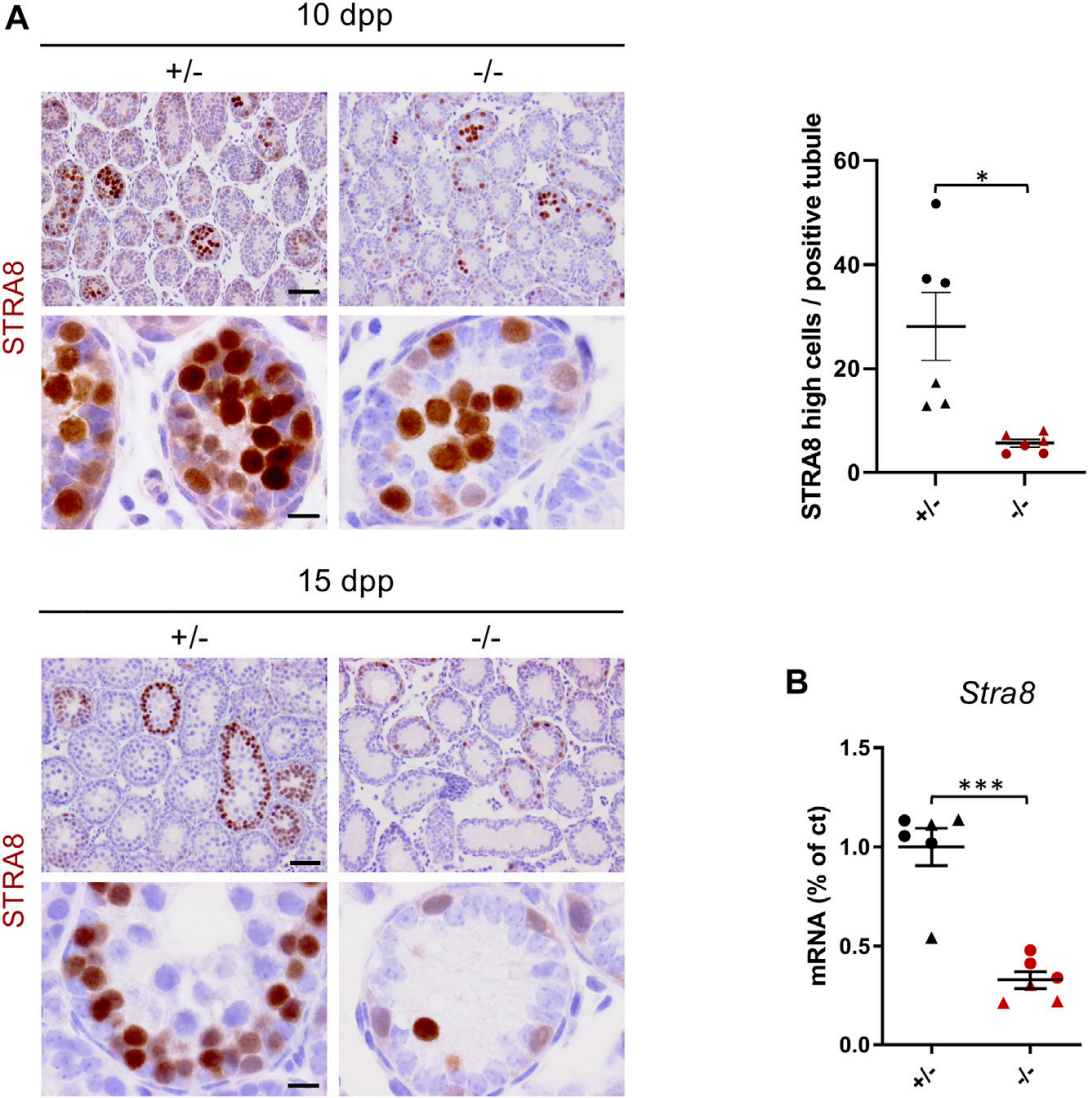
Meiotic entry is not impaired in *Cep250* ^*-/-*^ mutants. **(A)** Histological analysis of 10 dpp and 15 dpp testis sections stained for STRA8. Scale bar: 50 µm (upper panels) and 10 µm (lower panels). Quantification of the number of STRA8 high-positive cells per positive tubule is shown in the graph. Triangles represent 10 dpp values and circles represent 15 dpp values. Mean ± SEM. *p < 0.05 (two-way ANOVA). **(B)** *Stra8* mRNA expression levels were measured by RT-qPCR in the post-natal testes. Triangles represent 10 dpp values and circles represent 15 dpp values. *β-actin* was used as an endogenous reporter. Data are expressed as a percentage of the control expression level. Mean ± SEM. ***p < 0.001 (two-way ANOVA).

Despite a remarkable reduction in the population of differentiating spermatogonia, we still observed germ cells reaching the meiotic prophase I. However, we did not detect any meiotic metaphasic cells or post-meiotic germ cells, which suggested a second role of Cep250 in the regulation of meiotic prophase I progression. Accordingly, we next analyzed the mitosis-to-meiosis transition in *Cep250* ^*-/-*^ testes using STRA8 immunostaining (Figure 5A). At 10 dpp, we observed *Cep250* ^*-/-*^ tubules containing high STRA8-positive preleptotene spermatocytes, showing that spermatogonia that achieved differentiation entered meiosis. At 15 dpp we observed fewer preleptotene spermatocytes in the mutant testes than in the control testes. *Stra8* mRNA levels were significantly reduced in both 10 and 15 dpp mutant testes when compared to those in the controls (Figure 5B). As *Stra8* is specifically expressed in germ cells, we also normalized its expression with that of *Ddx4* in order to consider the variation in the global germ cell number and obtain an estimation of the proportion of germ cells that initiate meiosis. When *Stra8* expression was reported to that of *Ddx4,* it remained steady in mutants, indicating that a similar proportion of germ cells initiate meiosis at 10 and 15 dpp (Supplementary Figure S9).

### Cep250 is Essential for Meiotic Prophase I Progression

**FIGURE 6:**
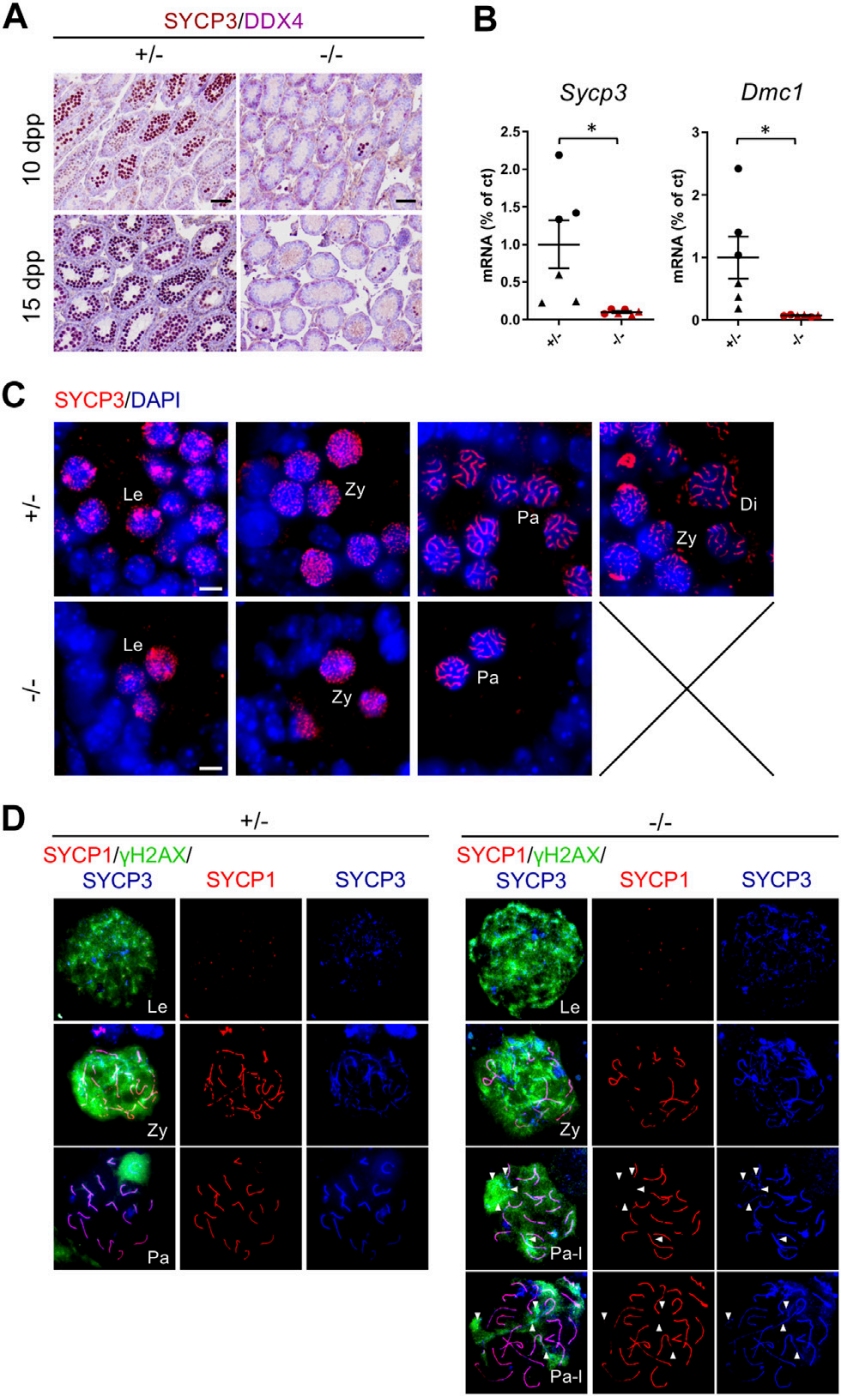
Meiotic progression is impaired in *Cep250* ^*-/-*^ mutants. **(A)** Histological analysis of 10 dpp and 15 dpp testis sections stained for the meiotic marker SYCP3 (brown) and the germ cell marker DDX4 (purple). Scale bar: 50 µm. **(B)** *Sycp3* and *Dmc1* mRNA expression levels were measured by RT-qPCR in the post-natal testes. Triangles represent 10 dpp values and circles represent 15 dpp values. *β-actin* was used as an endogenous reporter. Data are expressed as a percentage of the control. Mean ± SEM. *p < 0.05 (two-way ANOVA). **(C)** Immunofluorescence analysis of SYCP3 (red) in 15 dpp testis sections. DNA is stained with DAPI. Le: Leptotene; Zy: Zygotene; Pa: Pachytene; Di: Diplotene. Scale bar: 5 µm. **(D)** Co-immunolabelling of SYCP3 (blue), SYCP1 (red) and *γ*H2AX (green) on the spermatocyte chromosome spreads. Le: Leptotene; Zy: Zygotene; Pa: Pachytene; Pa-l: Pachytene-like. White arrowheads indicate asynapsis (SYCP3-stained axes, unstained for SYCP1).

As some germ cells were able to initiate meiosis in *Cep250* ^*-/-*^ testes, we monitored the progression of spermatocytes through prophase I. To this end, we stained the lateral element of the synaptonemal complex SYCP3, a marker of prophase I spermatocytes, which allowed us to recognize the different stages of prophase I (Figure 6). In the control 10 dpp testes, most tubules contained early meiotic prophase I spermatocytes at the leptotene and zygotene stages (Griswold, 2015). At 15 dpp, all tubules contained SYCP3-positive, and all the stages of prophase I could be visualized. In accordance with the reduction in germ cell population observed in the meiotic prophase I, we observed rare SYCP3-positive cells in *Cep250* ^*-/-*^ postnatal testes, both at 10 and 15 dpp (Figure 6A; Supplementary Figure S10). Consistently, mRNA levels of the meiotic markers *Sycp3* and *Dmc1* were significantly reduced in mutant testes compared to those in the controls (Figure 6B; Supplementary Figure S9). Interestingly, *Cep250* ^*-/-*^ testes fully lacked diplotene spermatocytes, indicating an arrest of prophase I at the pachytene stage (Figure 6C).

Based on these observations, we investigated meiotic prophase I events to identify defects that prevent prophase I progression (Figure 6D). Two major events occur during prophase I: Homologous chromosome synapsis and DSB formation and repair. Immunostaining of the synaptonemal complex and DSBs on the spermatocyte chromosome spreads allowed us to monitor these events. In wild-type testes, synaptonemal complex began forming during the leptotene stage, with lateral elements stained by SYCP3 beginning to assemble along the chromosome axes. At zygotene, synaptonemal central elements, stained by SYCP1, formed a zipper-like structure between the lateral elements of homologous chromosomes. Except for the sex chromosomes, other chromosomes were fully synapsed at the pachytene stage. In parallel, DSBs formed at the leptotene and zygotene stages were monitored using *γ*H2AX staining. At the pachytene stage, the DSBs were completely repaired and *γ*H2AX remains exclusively on the sex chromosomes. In *Cep250* ^*-/-*^ spermatocytes, DSBs formed normally at the leptotene and zygotene stages, and the homologous chromosomes initiated pairing (Figure 6D). At the pachytene-like stage, synapsing defects were observed, with some autosomal chromosomes stained for SYCP3 but not SYCP1, indicating a failure to complete synapsis. In addition, we observed the persistence of *γ*H2AX, potentially indicating defects in DSB repair.

Altogether, we observed an arrest of meiotic prophase I at the pachytene stage, likely due to synapsis defects and *γ*H2AX persistence in pachytene-like *Cep250* ^*-/-*^ spermatocytes.

### Fragmentation of Centrosomes

**FIGURE 7:**
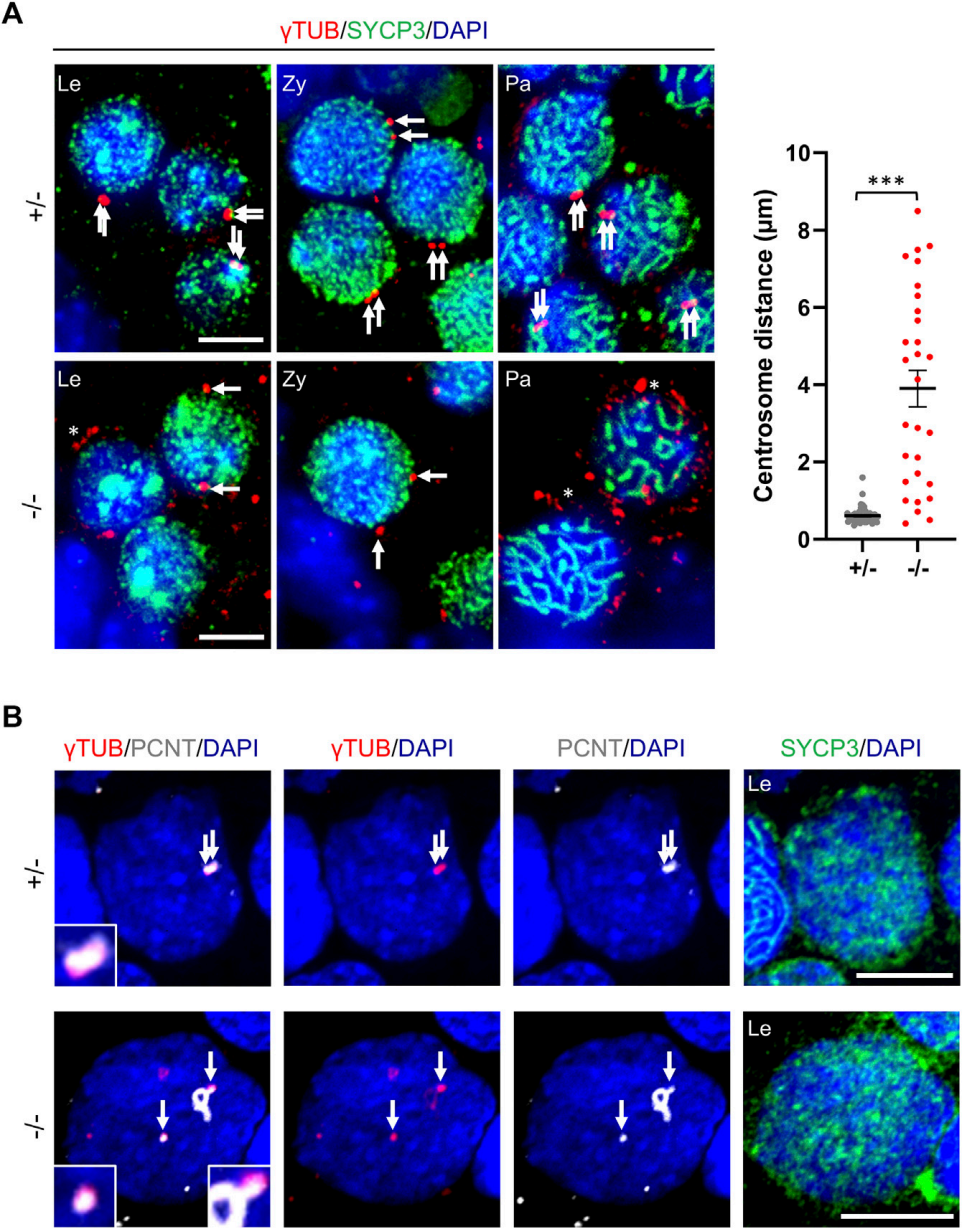
Centrosome cohesion is impaired in the absence of Cep250. **(A)** Immunofluorescence analysis of *γ*-tubulin (red) and SYCP3 (green) in the post-natal testis sections (15 dpp). DNA is stained with DAPI. Arrows indicate *γ*-tubulin pericentromeric material. Asterisks indicate fragmentation of *γ*-tubulin. Le: Leptotene; Zy: Zygotene; Pa: Pachytene. Scale bar: 5 µm. Quantification of the *γ*-tubulin foci distance is shown in the right panel. +/− *n* = 51 −/− *n* = 29; mean ± SEM. ***p < 0.001 *t*-test. **(B)** Immunofluorescence analysis of *γ*-tubulin (red), pericentrin (PCNT, gray) and SYCP3 (green) in post-natal testis cells. Magnification of *γ*-tubulin and pericentrin stained centrosomes are shown. Le: Leptotene. Scale bar: 10 µm.

Considering the centromeric localization of CEP250, we investigated the centrosome structure during prophase I. We immunolocalized *γ*-tubulin in the postnatal testes. At the beginning of prophase I, the two centrioles were linked together, appearing as two overlapping *γ*-tubulin spots in the control mice. In contrast, we detected two separated or fragmented *γ*-tubulin spots in the *Cep250* ^*-/-*^ early prophase I spermatocytes (Figure 7A). Pericentrin (PCNT) is another component of the pericentriolar material that behaved as *γ*-tubulin in control spermatocytes, forming two closely associated spots (Figure 7B). In *Cep250* ^*-/-*^ meiocytes, PCNT staining was found both as foci colocalizing with the distant *γ*-tubulin spots and in other filament-like structures. These observations indicate that CEP250 is mandatory for centrosome cohesion in male meiocytes, which is consistent with previous studies in other cell types (Mayor et al., 2000; Bahe et al., 2005; Floriot et al., 2015).

### CEP250 is Dispensable in Female Meiocytes

**FIGURE 8:**
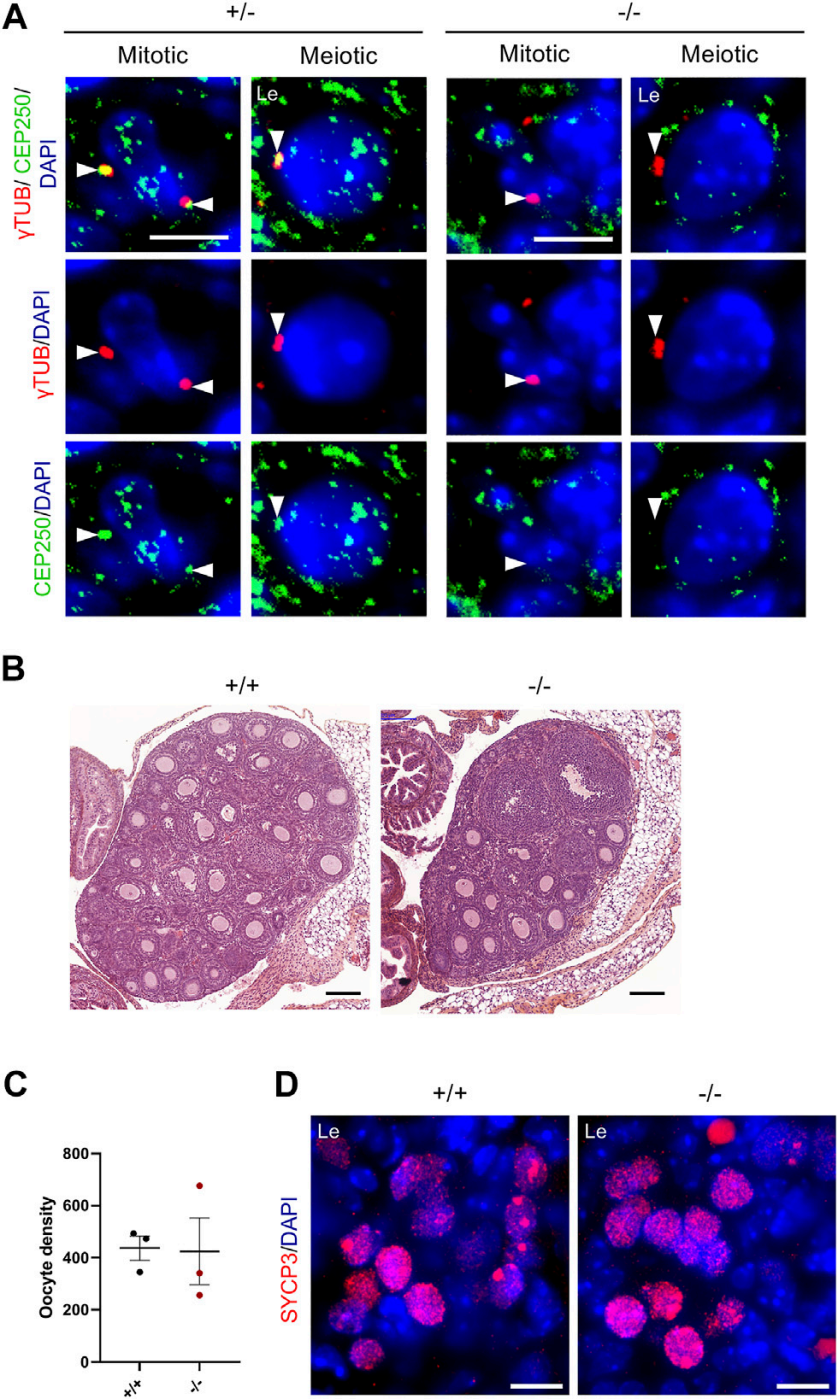
Cep250 is not mandatory during oogenesis. **(A)** Immunofluorescence staining of *γ*-tubulin (*γ*TUB, red) and CEP250 (green) in the control (+/−) and mutant (−/−) 15.5 days post-conception (dpc) foetal ovaries. DNA is stained with DAPI. White arrowheads indicate *γ*-tubulin-stained centrosomes. Note the absence of CEP250 in the centrosomes from mutant female germ cells. Le: Leptotene. Scale bar: 5 µm. **(B)** Hematoxylin/eosin-stained sections of the control (+/+) and mutant (−/−) adult ovaries. Scale bar: 200 µm. **(C)** Quantification of the oocyte density in post-natal ovaries (5–10 dpp). Mean ± SEM. **(D)** Immunofluorescence staining of SYCP3 (red) in the control (+/−) and mutant (−/−) 15.5 days post-conception (dpc) foetal ovaries. Le: Leptotene. Scale bar: 10 µm.

*Cep250* mRNA was steadily expressed in foetal and adult ovaries (Figure 1B). We detected CEP250 protein at the centrosomes of mitotic and meiotic germ cells in fetal wild-type ovaries but not in *Cep250* ^*-/-*^ ovaries (Figure 8A). In contrast to males, *Cep250* ^*-/-*^ females were fully fertile (Supplementary Table S4). Adult mutant ovaries had a normal size, and histological analysis showed a density of follicles equivalent to that of wild-type adult ovaries (Figure 8B). Accordingly, we observed a normal density of post-natal oocytes between 5 and 10 dpp (Figure 8C) and of SYCP3-positive germ cells in the mutant fetal ovaries (Figure 8D).

**FIGURE 9:**
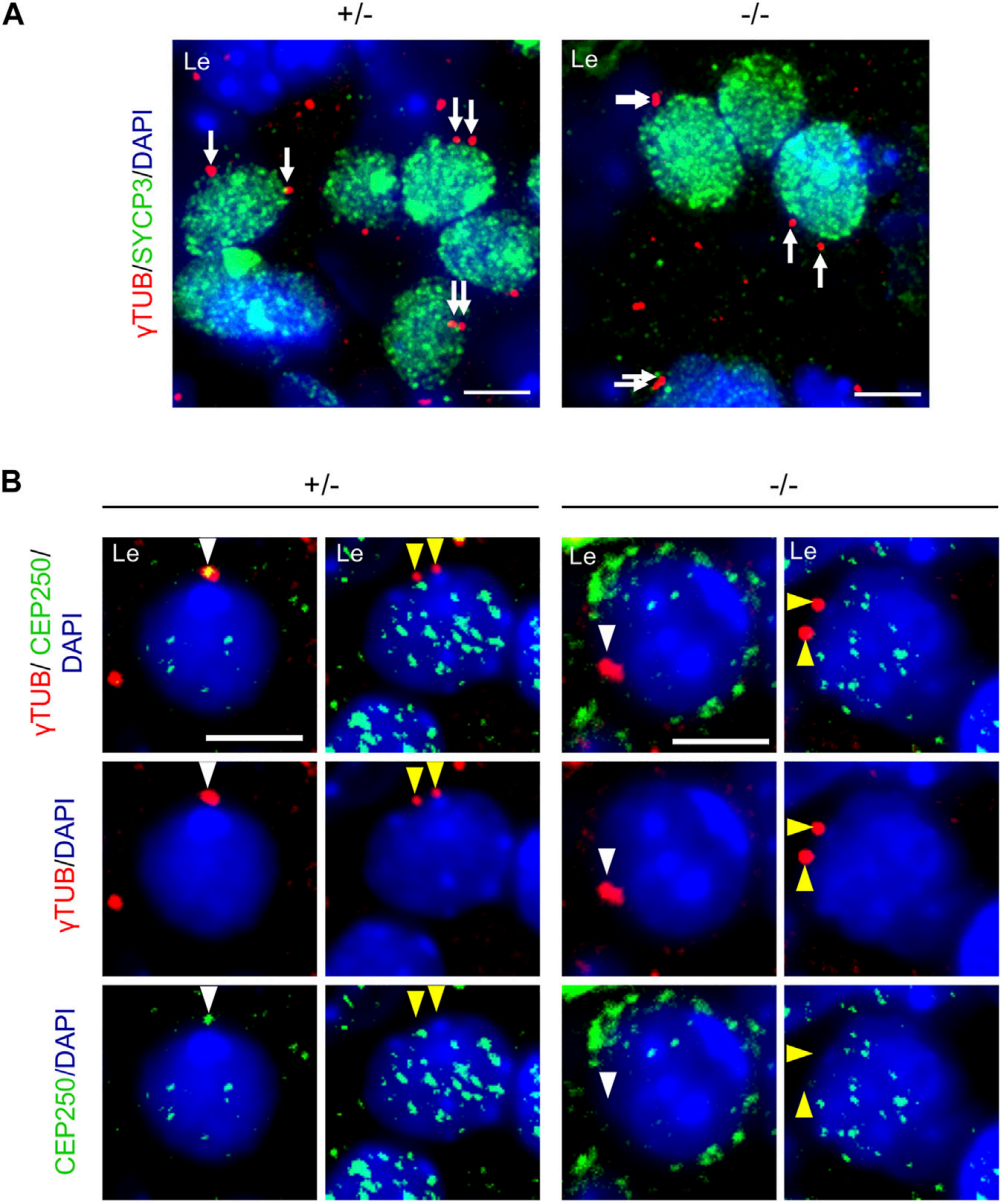
Female centrosomes display little cohesion at meiotic entry. **(A)** Immunofluorescence staining of *γ*-tubulin (*γ*TUB, red) and SYCP3 (green) in the control (+/−) and mutant (−/−) 15.5 days post-conception (dpc) foetal ovaries. DNA is stained with DAPI. White arrows indicate *γ*-tubulin. Le: Leptotene. Scale bar: 5 µm. **(B)** Immunofluorescence staining of *γ*-tubulin (*γ*TUB, red) and CEP250 (green) in the control (+/−) and mutant (−/−) 15.5 days post-conception (dpc) foetal ovaries. DNA is stained with DAPI. White arrowheads indicate cohesive *γ*-tubulin foci. Yellow arrowheads indicate separated *γ*-tubulin foci. Note that CEP250 is not present in *γ*-tubulin-stained area in the control oocytes when these are separated. Le: Leptotene. Scale bar: 5 µm.

To assess the difference observed between male and female meiocytes in response to *Cep250* invalidation, we examined centrosomes in wild-type meiotic oocytes at the beginning of prophase I. Interestingly, we could distinguish two different cell populations of germ cells at early stages of prophase I in the ovaries. Some early prophase I oocytes possessed two paired *γ*-tubulin foci, with CEP250 located between them (Figures 9A,B) as in spermatocytes (Figure 7A). In other oocytes, *γ*-tubulin foci were separated at the leptotene stage; in this case, CEP250 was not observed (Figures 9A,B). Both the populations of oocytes, with paired or separated foci, were also observed in *Cep250* ^*-/-*^ ovaries.

These results suggest a sexual dimorphism in the control of the meiotic centrosome structure in mice. A precocious separation of centrosomes in males leads to spermatogenesis defects and germ cell death, whereas in females, germ cells normally progress through oogenesis even if the centrosomes split up.

## Discussion

Altogether, our data indicate that CEP250 is essential for maintaining centrosome cohesion in male germ cells. The invalidation of *Cep250* induces centrosome precocious separation at the early stages of spermatogenesis, resulting in male infertility.

Our analysis of spermatogenesis in *Cep250* ^*-/-*^ mice revealed that two main steps were primarily affected: The differentiation of spermatogonia and meiotic prophase I progression. This is in accordance with the high expression of *Cep250* in the postnatal testis when spermatogenesis is initiated. The enhanced mitotic amplification of germ cells is a hallmark of male gametogenesis, allowing the production of numerous gametes. The spermatogonial stem cells display a slow proliferation rate, whereas high mitotic activity is observed when the spermatogonial cells differentiate. These active divisions can easily be considered as requiring efficient centrosomes. In this regard, it is interesting to note that while we did not detect any major defects in the spermatogonial stem cells, differentiating spermatogonia were mostly affected and lost in the absence of *Cep250*. In contrast, *Cep250* ^-/-^ meiosis was arrested during prophase I; thus prior to meiotic division. In this regard, the meiotic arrest we observed related to precocious centriole separation or centrosome fragmentation was unexpected. Spermatocytes appear to rely on a higher centriolar cohesion to complete meiosis I. Of note, we cannot rule out the possibility of a role of *Cep250* in post-meiotic cells.

*Cep250* invalidation triggers a lack of centrosome cohesion in male meiocytes, as evidenced by monitoring the distance between *γ*-tubulin foci, a marker of the pericentriolar material. Future work should also monitor centriole and fiber markers (e.g., CENTRIN1 or ROOTLETIN) to further define this centrosomal defect. Nonetheless, the lack of centrosome cohesion we observed is consistent with CEP250 being part of the fibers linking the paternal centrioles after the duplication of the centrosome (Mayor et al., 2000) and with its inhibition triggering centriole separation in cell lines (Bahe et al., 2005; Flanagan et al., 2017). Whether such a defect also occurs in the late differentiated spermatogonia was difficult to determine in this study, due to small cells grouped together that did not allow a faithful identification of the origin of centrosomes in sections. Future studies using testicular cell spreads could address this issue. However, based on the role of CEP250, it can be assumed that centrosomes are similarly affected in the spermatogonia. Interestingly, we observed *γ*H2AX staining in metaphasic spermatogonia lacking *Cep250*. This indicates the likely presence of unrepaired DNA DSBs in these cells, which can explain the apoptosis and loss of a large fraction of these cells. Interestingly, defects in the centrosome linker have been recently proposed to cause genetic instability and DNA damage in other cells (Sivasubramaniam et al., 2008; Staples et al., 2012; Mullee and Morrison, 2016). Several proteins of the DNA damage response (DDR) proteins, including the ATM, ATR, CHK1, CHK2, and BRCA1, are also found at centrosomes (Zhang et al., 2007; Shimada and Komatsu, 2009). Therefore, it is tempting to propose that the altered centrosomes in *Cep250* ^*-/-*^ spermatogonia may indirectly cause genetic instability. Another view may be that the mis-segregated chromosomes due to centrosome dysfunction may suffer from extensive DNA damage, as recently suggested (Pihan, 2013).

The need for centrosome cohesion during meiotic prophase I is male-specific, as this was not observed in fetal oocytes, and the *Cep250* ^*-/-*^ females were fully fertile. We speculate that the endogenous lack of cohesion in some oocytes was related to centrosome loss a female-specific feature phenomenon during meiosis prophase I. Indeed, the meiotic division in mammalian oocytes is known to be acentrosomal (Dumont and Desai, 2012). In contrast, male meiotic divisions rely on the establishment of regular spindles originating from two MTOCs (i.e., centrosomes). Whether the lack of centrosome cohesion in wild-type fetal oocytes is due to the absence of CEP250 warrants further exploration. Of note, CEP250 was found later at acentriolar MTOC during oocyte maturation (Clift and Schuh, 2015; Little and Jordan, 2020). Nonetheless, this male-specific requirement for centrosome cohesion during early meiotic prophase I suitably fits the sex-specific characteristics of gametogenesis.

Surprisingly, spermatocyte death occurred prior to the first meiotic division, at the pachytene-like stage, and no diplotene spermatocytes were observed in *Cep250* mutant mice. During the male meiotic prophase, two cohesive centrioles were observed during the leptotene stage. These centrosomes start duplicating at the zygotene stage and split up at the diplotene stage when they start migrating to opposite poles of the cell (Alfaro et al., 2021; Wellard et al., 2021). Our present study revealed that precocious separation of the centriole triggered meiotic arrest, likely due to the unrepaired DSBs. Indeed, in *Cep250* ^*-/-*^ spermatocytes, high levels of *γ*H2AX were observed even in well-synapsed chromosomes. This indicates that meiotic DSB repair requires centrosome cohesion. This effect of centrosomal alteration outside the nucleus may appear odd in the absence of cell division. It may also be proposed that centrosomes regulate the activity or expression of DDR proteins. In this regard, the sharp decrease in *Dmc1* expression observed in Cep250^-/-^ testes is of interest, as DMC1 is a recombinase crucial for DNA repair during meiotic homologous recombination. It may also be considered that centrosomes orchestrate the microtubule network in the cell. In meiotic cells, chromosome movements have been proposed to help repair meiotic breaks. It should be pointed out that a reduction in centrosome-mediated chromosome movements has already been proposed to underlie recombination failure during meiosis in the case of the invalidation of Cep63, which also causes a lack of centrosome cohesion (Marjanović et al., 2015). Nonetheless, in both cases, it is expected that similar defects would be observed during female meiosis when the centrosomes naturally split up and degenerate. A tentative hypothesis is the existence of a new meiotic checkpoint in males that ensures centrosome integrity prior to meiotic divisions. Further studies should address signaling related to centrosomal dysfunction and meiotic DSB repair.

In conclusion, we revealed a major function of CEP250 during mouse spermatogenesis, with no other gross alteration of physiological functions. Our previous study provides robust evidence that spontaneous mutation of this centrosome linker causes a Seckel-like syndrome in cattle (Floriot et al., 2015). This phenotypic difference may be due to species specificity. On the other hand, the poor representation of boars in modern cattle breeding prevented the examination of mutant male fertility in this species. Ultimately, CEP250 is expected to function in all cells as a centriole anchor; thus, it is surprising that its invalidation specifically alters the male germline. Whether subtle phenotypic alterations exist in other tissues is currently under investigation.

## Data Availability

The datasets presented in this study can be found in online repositories. The names of the repository/repositories and accession number(s) can be found below: NCBI GenBank BankIt, accession numbers: BankIt2498187 CEP2 OK076696 and BankIt2498187 CEP3 0K076697.
