# Supplementary material for: CEP250 is Required for Maintaining Centrosome Cohesion in the Germline and Fertility in Male Mice
Source: Front Cell Dev Biol. 2022 Jan 19;9:754054. doi: 10.3389/fcell.2021.754054 (PMC8809461; doi:10.3389/fcell.2021.754054)
Supplement: Supplementary file 1 [file Table1.PDF]

S1 Table: Male fertility phenotype

Genotype	Number of fertile/ tested males (n)
+/-	22/22
-/-	0/4

S2 Table: Female fertility

Genotypes of females	Number of litters	Genotypes of males	Means ± SD
+/+	12	+/-	6.50 ± 1.88
+/-	87	+/-	6.79 ± 1.98
-/-	22	+/-	5.59 ± 1.68

S3 Table: List of all primers used in the study

Name	Species	Applications	Forward	Reverse
aSMA	Mouse	RT-qPCR	5′-AACGCTTCCGCTGCCC-3′	5′-GGAGCCACCGATCCAG-3′
Actb	Mouse	RT-qPCR	5′-GCCCTGAGGCTCTTTTCCAG-3′	5′-TGCCACAGGATTCCATACCC-3′
Cep250_339	Mouse	PCR	5′-GGAGGTGAACACCGAGCTT-3′	5′-ACCTGCCAACAAAGCCTAGA-3′
Cep250_1018	Mouse	RT-qPCR	5′-CAGCAGGTGATCAAGGCTATAACC-3′	5′-CAAACTGGGAAAGGCCATTACA-3'
Cep250_3665	Mouse	RT-PCR	5′-GGCGACTCTGCTCCTACCTT-3′	5′-CCCTTCCCACTTGGATTTCT-3′
Ddx4	Mouse	RT-qPCR	5′-GAAGAAATCCAGAGGTTGGC-3′	5′-GAAGGATCGTCTGTCTGAACA-3′
Dmc1	Mouse	RT-qPCR	5′-CCCTCTGTGTGACAGCTCAA-3′	5′-GGTCAGCAATGTCCCGAAG-3′
Gapdh	Mouse	RT-PCR	5′-GCATCTCCCTCACAATTTCCA-3′	5′-TGCAGCGAACTTTATTGATGGT-3′
Gata4	Mouse	RT-qPCR	5′-CCCCAATCTCGATATGTTTG-3′	5′-CCCCACAATTGACACACTCT-3′
Hsd3b	Mouse	RT-qPCR	5′-TTCCAGCCTTCATCTTCTC-3′	5′-TCTTCCTCGTTGCCATTC-3′
Kit	Mouse	RT-qPCR	5′-AATGGCCTCACGAGTTCTAT-3′	5′-ATGGAGTTCACGGATGTAGA-3′
Sall4	Mouse	RT-qPCR	5′-GAGCTGCCGCGTTGACA-3′	5′-TGTGCTCGGATAAATGTTGGAG-3′
Sox9	Mouse	RT-qPCR	5′-CCTTCCTCACTACAGCCCCT-3′	5′-GTCAGCGTAGTCGTATTGCG-3′
Stra8	Mouse	RT-qPCR	5′-TGAAGCTCAAAGCATCCTTCAA-3′	5′-CTAAGCTGTTGGGATTCCCATC-3′
Sycp3	Mouse	RT-qPCR	5′-AAAGAAATGGCTATGTTGCAAAAA-3′	5′-TTGCCACTCCTTGCTGCTGA-3′

S4 Table: List of the antibodies used in the study

Name	Company	Reference	Host species	Mono/poly	Concentration	Applications
γH2AX	Abcam	ab11174	Rabbit	Poly	1/1000	IF
γH2AX	Millipore	05-636	Mouse	Mono	1/1000	IF
γ-tubulin	Sigma	T3539	Rabbit	Poly	1/200	IF
cleaved caspase-3	Cell Signaling	9661S	Rabbit	Poly	1/200	IHC
CEP250	Santa-Cruz	SC-390540	Mouse	Mono	1/50	IF
DDX4	Abcam	ab27591	Mouse	Mono	1/200	IHC - IF
DDX4	Abcam	ab13840	Rabbit	Poly	1/200	IHC - IF
Ki67	BD Pharmingen	550609	Mouse	Mono	1/200	IF
GATA1	Cell Signaling	3535S	Rabbit	Poly	1/200	IHC
GATA4	Santa Cruz	SC-1237	Goat	Poly	1/200	IHC
PCNT	Abcam	ab220784	Rabbit	Poly	1/200	IF
pH3	Cell Signaling	9706S	Mouse	Mono	1/200	IF
PLZF	Santa Cruz	SC-22839	Rabbit	Poly	1/50	IF
STRA8	Abcam	ab49602	Rabbit	Poly	1/1000	IHC
SYCP1	Abcam	ab15090	Rabbit	Poly	1/200	IF
SYCP3	Novus	NB300-232	Rabbit	Poly	1/500	IF
SYCP3	Abcam	ab97672	Mouse	Mono	1/500	IF
SYCP3	Home made		Guinea Pig	Poly	1/200	IF
Alexa488 anti-mouse	Life technologies	A21202	Donkey	Poly	1/00	IF
Alexa594 anti-mouse	Life technologies	A21203	Donkey	Poly	1/500	IF
Alexa488 anti-rabbit	Life technologies	A21206	Donkey	Poly	1/500	IF
Alexa594 anti-rabbit	Life technologies	A21207	Donkey	Poly	1/500	IF
Alexa350 anti-guinea-pig	Life technologies	SA5-10093	Donkey	Poly	1/500	IF
