# Supplementary material for: CEP250 is Required for Maintaining Centrosome Cohesion in the Germline and Fertility in Male Mice
Source: Front Cell Dev Biol. 2022 Jan 19;9:754054. doi: 10.3389/fcell.2021.754054 (PMC8809461; doi:10.3389/fcell.2021.754054)
Supplement: Supplementary file 2 [file Presentation1.PDF]

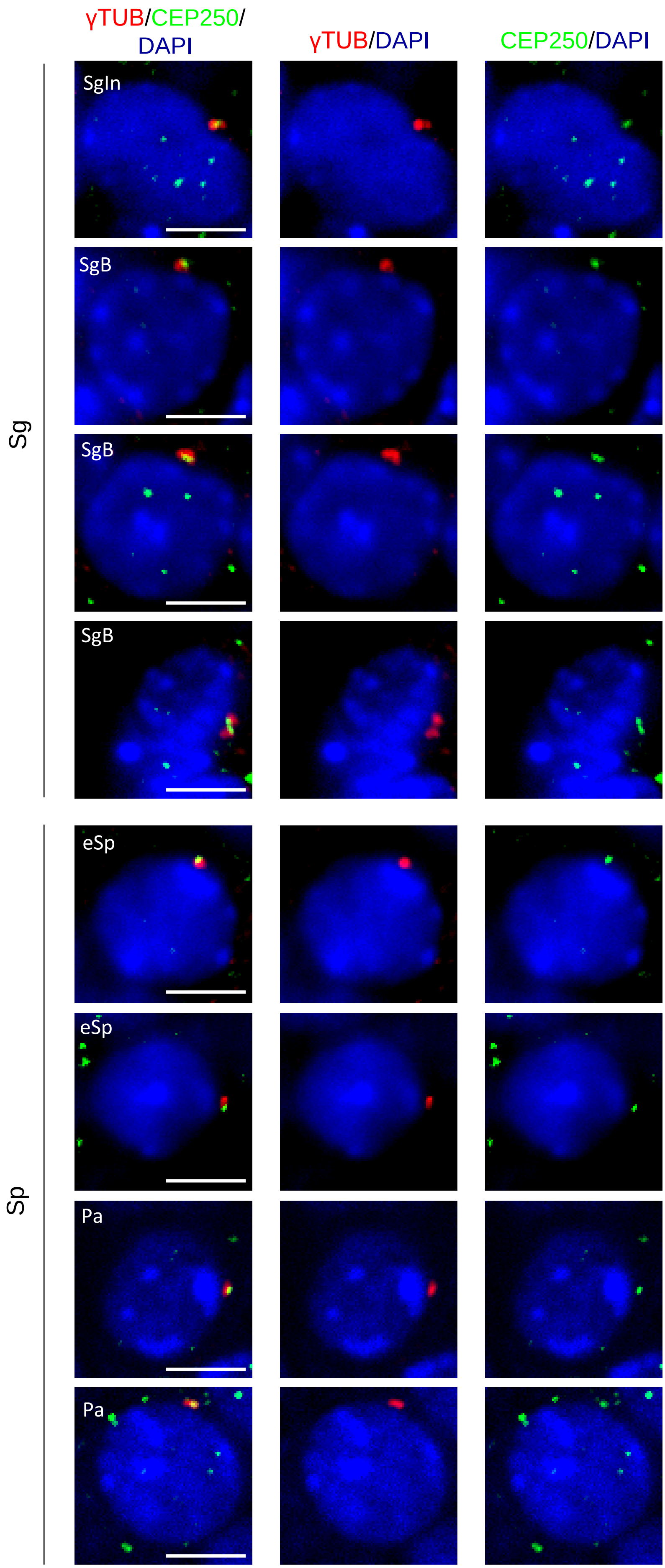
γTUB/CEP250/
DAPI
γTUB/DAPI
CEP250/DAPI
Sg
Sp
SgIn
SgB
SgB
SgB
eSp
eSp
Pa
Pa

S1 Figure.

A

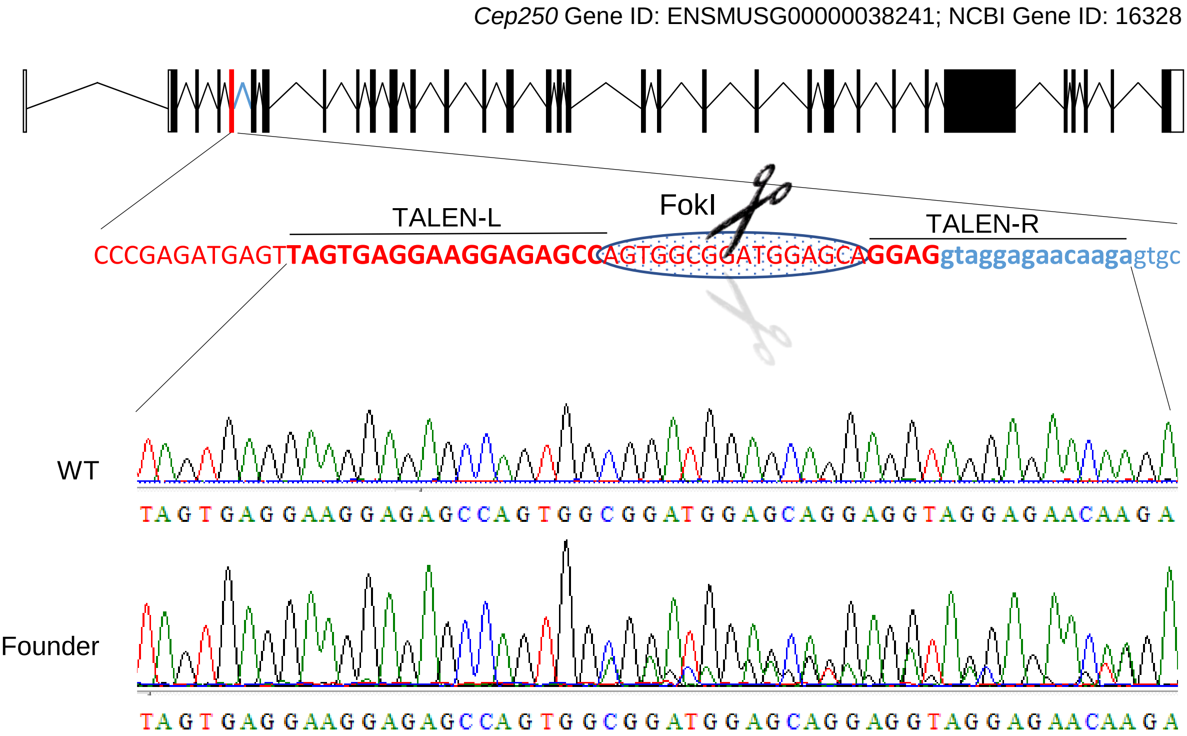

B

WT TAGTGAGGAAGGAGAGCCAGTGGCGGATGGAGCAGGAGgtaggagaacaaga

Founder TAGTGAGGAAGGAGAGCCAGTGG-----AGAGCAGGAGgtaggagaacaaga -6+1 bp

C

WT LVRKESQWRMEQEFFKGYLRGEHGRLLNLWREVVTFRRHFLKMKSATD... (160 above M)

Founder LVRKESQWRAGVLQGLFER* p.Met160Alafs*11

S2 Figure.

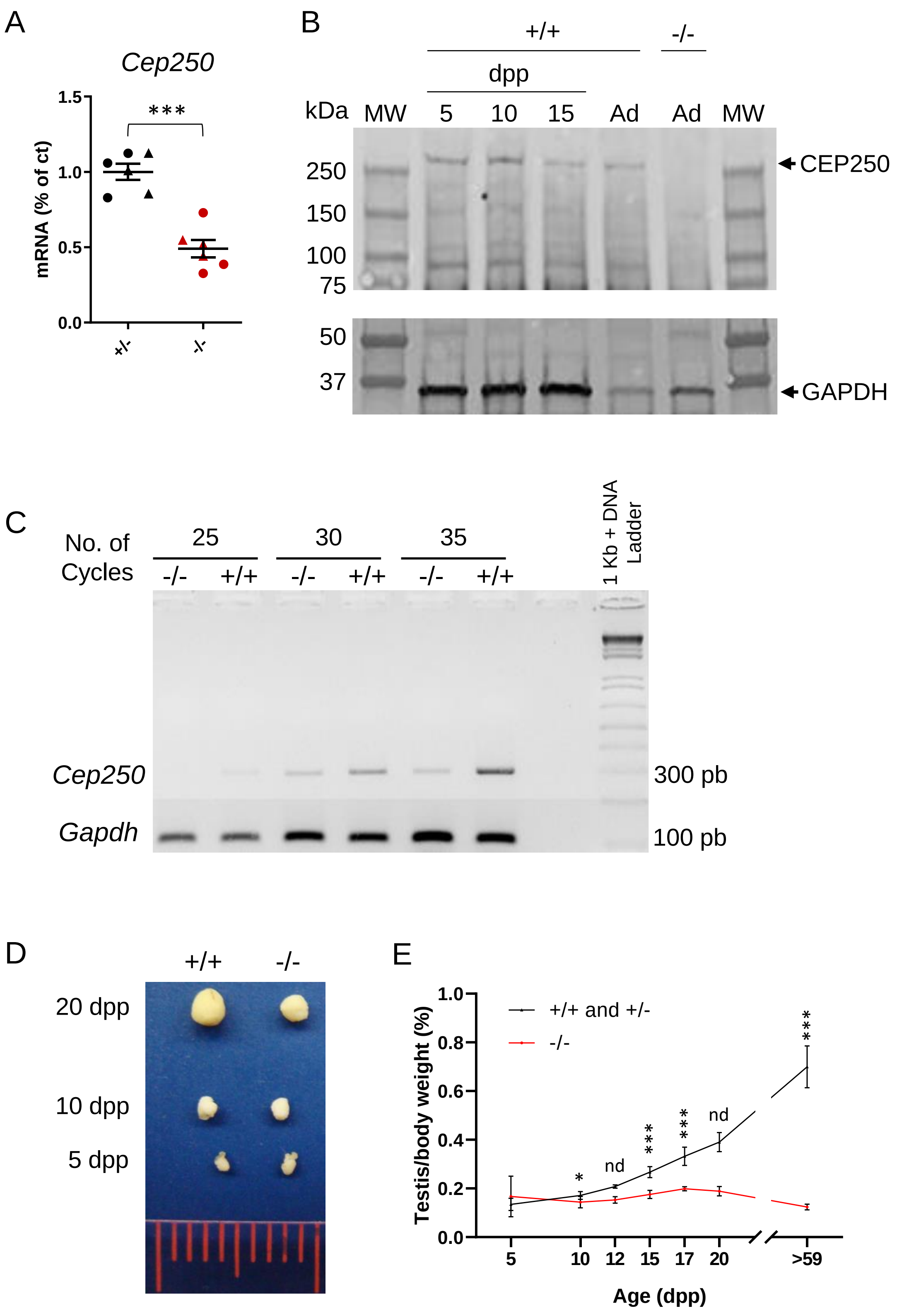

A
Cep250
mRNA (% of ct)
1.5
1.0
0.5
0.0
***
+/-
-/-
B
+/+
-/-
dpp
kDa
MW
5
10
15
Ad
Ad
MW
250
150
100
75
50
37
CEP250
GAPDH
C
No. of Cycles
25
30
35
-/-
+/+
-/-
+/+
-/-
+/+
1 Kb + DNA Ladder
Cep250
Gapdh
300 pb
100 pb
D
+/+
-/-
20 dpp
10 dpp
5 dpp
E
Testis/body weight (%)
1.0
0.8
0.6
0.4
0.2
0.0
+/+ and +/-
-/-
*
nd
***
***
nd
***
5
10
12
15
17
20
>59
Age (dpp)

S3 Figure.

A

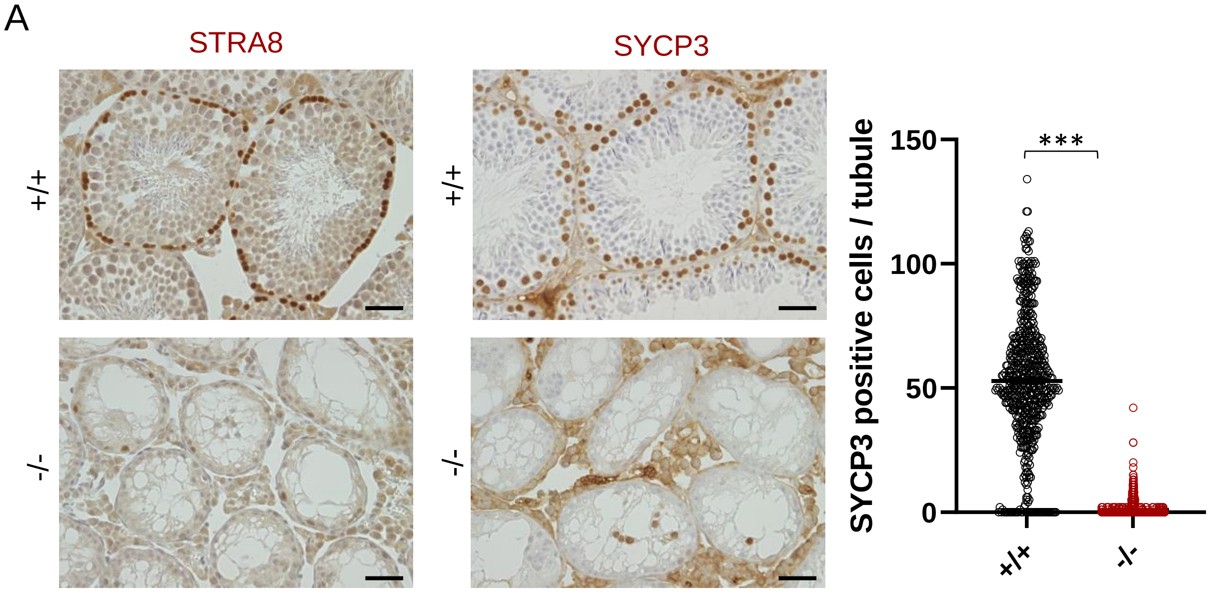

STRA8
SYCP3
+/+
-/-
+/+
-/-
SYCP3 positive cells / tubule
150
100
50
0
***
+/+
-/-

B

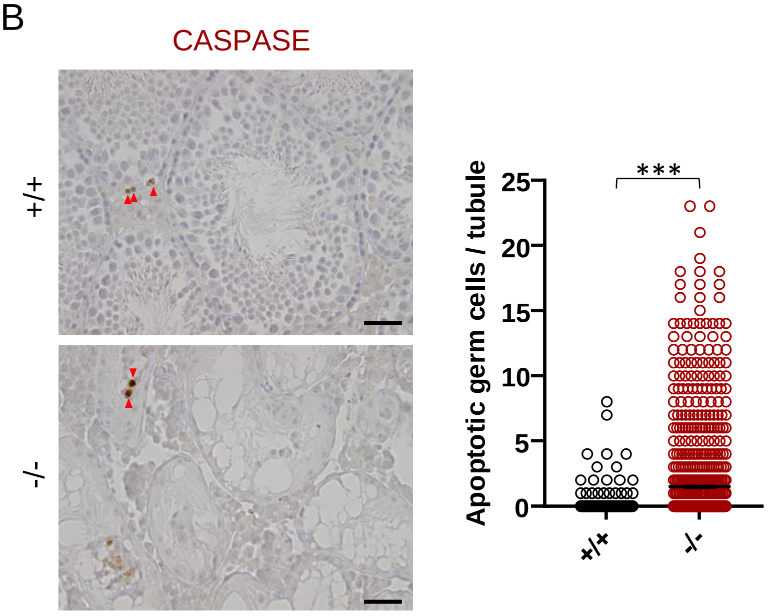

CASPASE
+/+
-/-
Apoptotic germ cells / tubule
25
20
15
10
5
0
***
+/+
-/-

S4 Figure.

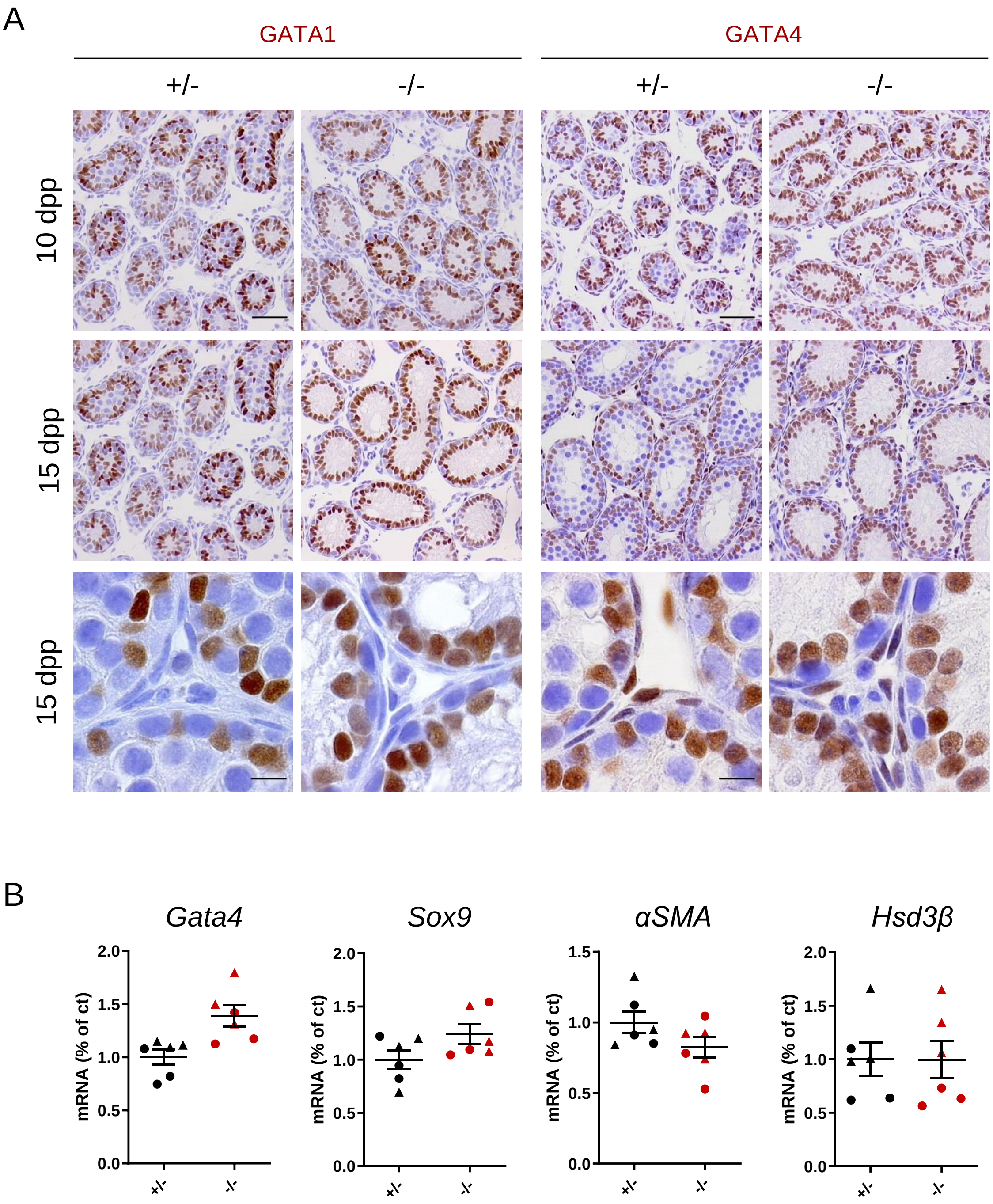

A
GATA1
GATA4
+/-
-/-
+/-
-/-
10 dpp
15 dpp
15 dpp
B
Gata4
Sox9
αSMA
Hsd3β
mRNA (% of ct)
2.0
1.5
1.0
0.5
0.0
+/-
-/-

S5 Figure.

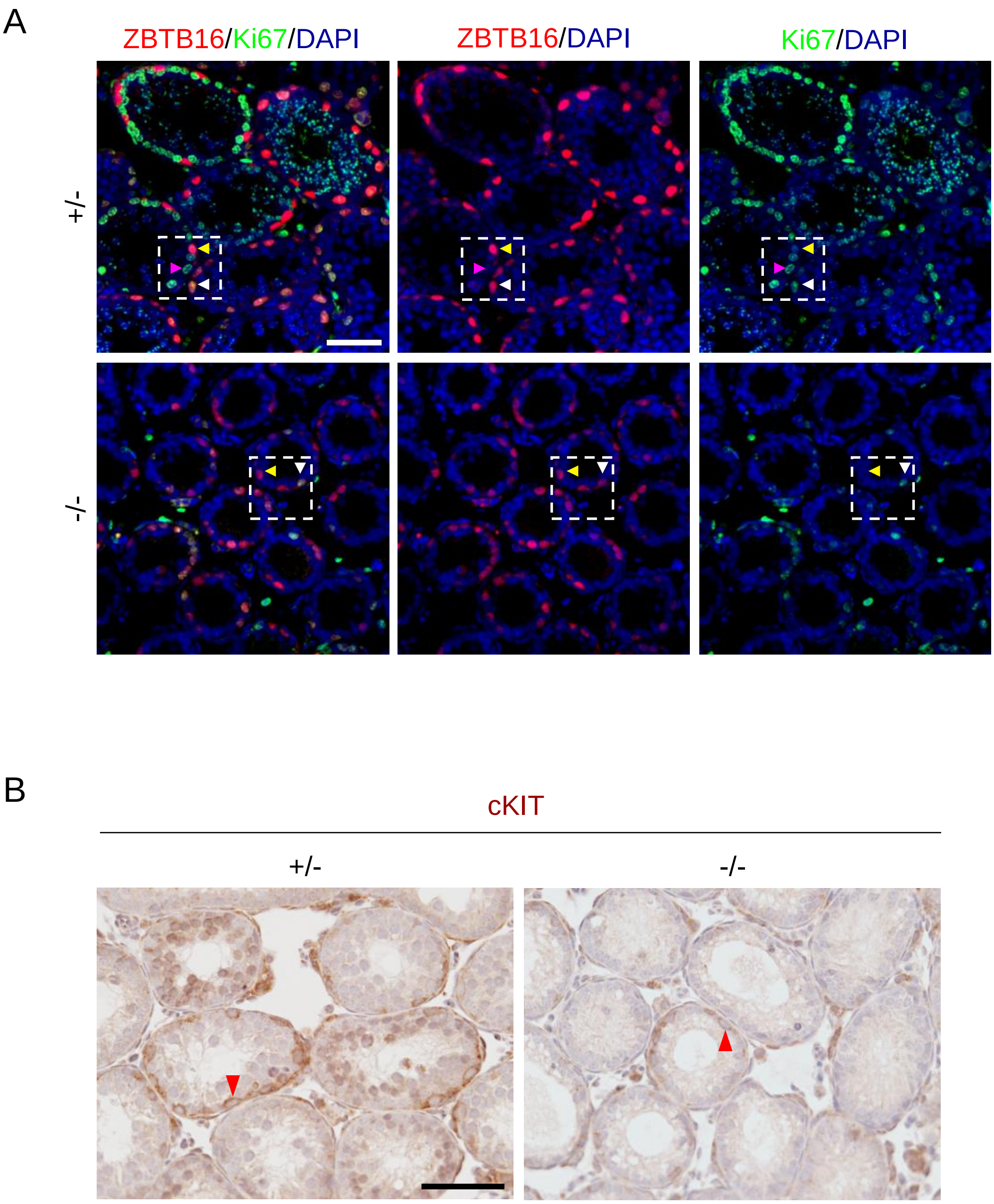

S6 Figure.

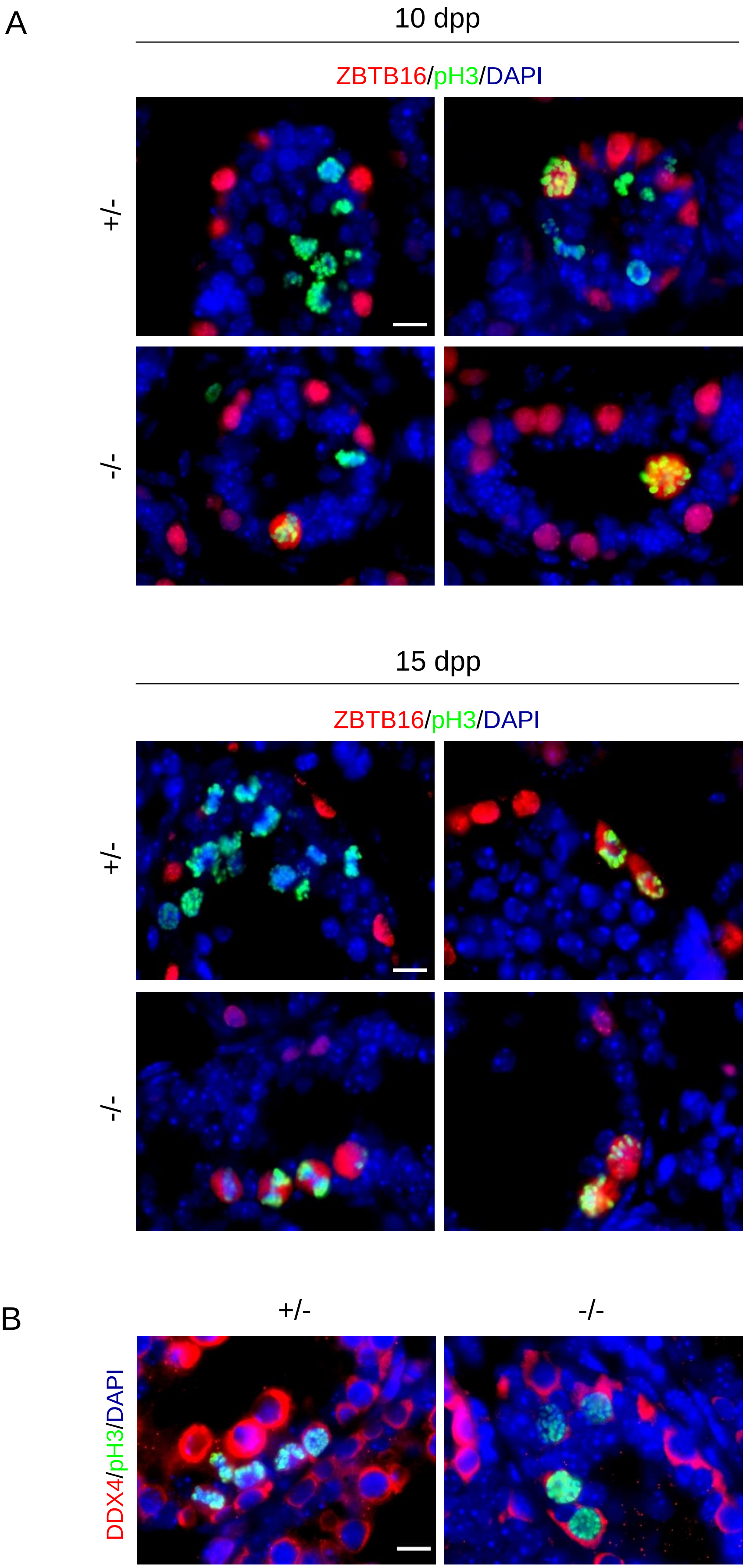

S7 Figure.

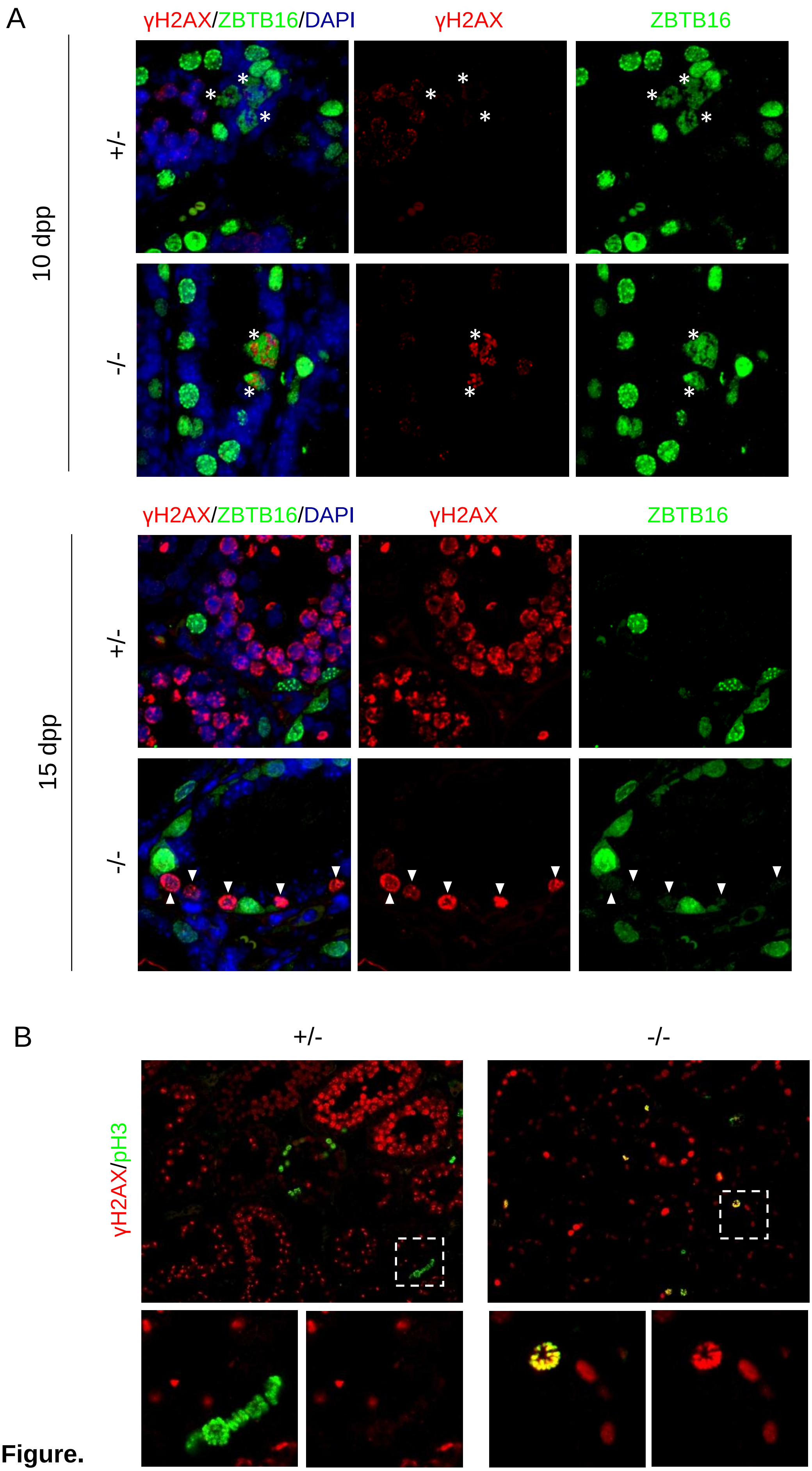

S8 Figure.

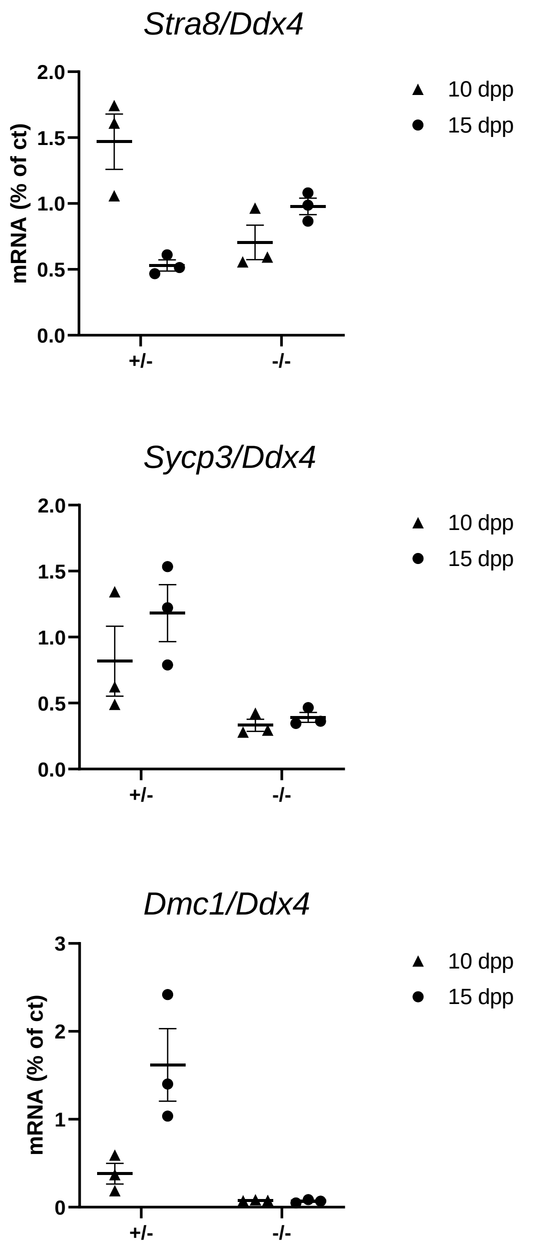
Stra8/Ddx4
mRNA (% of ct)
2.0
1.5
1.0
0.5
0.0
+/-
-/-
10 dpp
15 dpp
Sycp3/Ddx4
2.0
1.5
1.0
0.5
0.0
+/-
-/-
10 dpp
15 dpp
Dmc1/Ddx4
mRNA (% of ct)
3
2
1
0
+/-
-/-
10 dpp
15 dpp

S9 Figure.

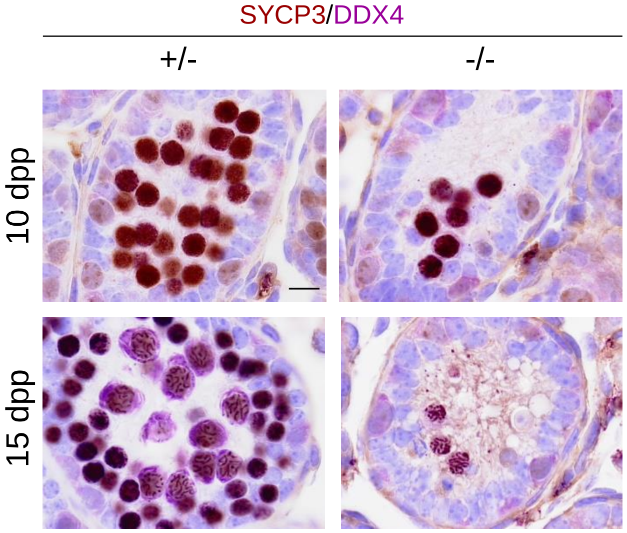

S10 Figure.
