# Supplementary material for: CEP250 is Required for Maintaining Centrosome Cohesion in the Germline and Fertility in Male Mice
Source: Front Cell Dev Biol. 2022 Jan 19;9:754054. doi: 10.3389/fcell.2021.754054 (PMC8809461; doi:10.3389/fcell.2021.754054)
Supplement: Supplementary file 3 [file DataSheet1.DOCX]

S1 Figure.

Immunofluorescence staining of γ-tubulin (γTUB, red) and CEP250 (green) in spermatogonia (Sg) and spermatocytes (Sp). SgIn: Intermediate spermatogonia; SgB: B spermatogonia; eSp: early prophase spermatocytes (Leptonema to early pachynema); Pa: mid-pachytene spermatocytes. DNA is stained with DAPI. Scale bar: 5 µm.

S2 Figure.

(A) Schematic of mouse *Cep250* gene, Sequence Map Chr2:155956458-155998900 bp, + strand (GRCm38p.2, C57BL/6J). Focus on the gDNA at the end of exon 5 (blue) and start of intron 5 (red) showing the target sites of TALENs in the mouse CEP250 locus. Exon sequences are in uppercases and intron sequences are in lowercases. Oval shape with scissors indicate *FokI* nuclease cutting site. Blue and red bold sequences indicate the left and right binding sites of CEP250-TALEN.

Chromatograms of the obtained sequences from Wild Type (WT) or founder animals by Sanger sequencing after PCR.

(B) Deduced allelic sequence information obtained from WT or founder animals. Nucleotide deletions (-) and insertion (A) are indicated in brown.

(C) Wild-type (WT) sequence of CEP250 protein is shown on the top. Predicted mutated founder amino acid sequence is indicated below. Mutated amino acids are highlighted in brown. Stop nonsense codon is showns as *.

S3 Figure.

(A) *Cep250* mRNA expression levels were measured by RT-qPCR in post-natal testes. Triangles represent 10 dpp values and circles represent 15 dpp values. *β-actin* was used as endogenous reporter. Data are expressed as percentage of the control expression level. Mean ± SEM. ***p < 0.001 (two-way ANOVA).

(B) Western Blot of control +/+ post-natal (5, 10, 15 dpp) and adult testis and mutant -/- adult testis proteins. An antibody against human centrosomal protein CEP250, targeting the N-term domain of the protein (Santa Cruz; Sc390540) was used. GAPDH was used as a control. Molecular weight markers (MW) are shown.

(C) Semi quantitative RT-PCR expression analysis of CEP250 genes in kidney of control +/+ and mutant -/- mice. Housekeeping GAPDH gene was used as endogenous reporter. Molecular weight markers (pb) are indicated (right margin). Genotypes and number of PCR cycles are indicated on the top.

(D) Photographies of control +/+ and mutant -/- post-natal testes of indicated developmental ages (5, 10, 20 days post partum, dpp).

(E) Analysis of testis weight/body weight from 5 to >59 day-old control (+/+ and +/-) and homozygous mutant (-/-) mice. Values correspond to mean ± SD, n = 4 to 37 excepted for +/+ and +/- at 12 dpp and -/- at 17 dpp (n = 2). nd, not determined; ***p < 0.001 (two-way ANOVA).

S4 Figure.

(A) Histological analysis of adult testis sections stained for STRA8 (brown, left panel) or SYCP3 (brown, right panel). Quantification of the number of SYCP3-positive germ cells per tubule is shown in the right panel. Mean ± SEM. ***p < 0.001 (two-way ANOVA).

(B) Histological analysis of adult testis sections stained for the apoptotic marker cleaved caspase 3 (brown). Red arrowheads denote the germ cells stained for the cleaved caspase 3. Scale bar: 40 µm. Quantification of the number of apoptotic germ cells per tubule is shown in the right panel. Mean ± SEM. ***p < 0.001 (two-way ANOVA).

S5 Figure.

(A) Histological analysis of 10 dpp and 15 dpp testis sections stained for the somatic Sertoli cell marker GATA1 or Sertoli and Leydig cell marker GATA4. Scale bar: 50 µm and 10 µm respectively for upper and lower images.

(B) mRNA expression levels of different somatic markers were measured by RT-qPCR in post-natal testes. Triangles represent 10 dpp values and circles represent 15 dpp values. *β-actin* was used as an endogenous reporter. Data are expressed as a percentage of the control. Mean ± SEM.

S6 Figure.

(A) Immunofluorescence analysis of the spermatogonia cell markers ZBTB16 (red) and Ki67 (green) in 15 dpp testis sections. DNA is stained with DAPI. Yellow arrowheads indicate spermatogonia stained exclusively for ZBTB16 (ZBTB16+Ki67-); white arrowheads indicate spermatogonia stained for both ZBTB16 and Ki67 (ZBTB16+Ki67+); magenta arrowheads indicate germ cells stained exclusively for Ki67 (ZBTB16-Ki67+). Dashed squares represent magnified area shown in Fig. 4D. Scale bar: 50 µm.

(B) Histological analysis of 15 dpp testis sections stained for differentiating spermatogonia cell marker cKIT (brown). Red arrowheads denote spermatogonia stained for cKIT. Scale bar: 50 µm.

S7 Figure.

(A) Immunofluorescence analysis of the spermatogonial cell marker ZBTB16 (red) and metaphase cell marker pH3 (green) in 10 dpp and 15 dpp testis sections. DNA is stained with DAPI. Scale bar: 10 µm.

(B) Immunofluorescence analysis of the germ cell marker DDX4 (red) and metaphase cell marker pH3 (green). DNA is stained with DAPI. DDX4 staining indicates the germ cell identity of metaphase cells. Scale bar: 10 µm.

S8 Figure.

(A) Immunofluorescence analysis of the DSBs marker γH2AX (red) and spermatogonial cell marker ZBTB16 (green) in 10 dpp and 15 dpp testis sections. DNA is stained with DAPI. Asterisks denote the metaphase cells. White arrowheads indicate differentiating spermatogonia stained for γH2AX and faintly stained for ZBTB16.

(B) Immunofluorescence analysis of DSB marker γH2AX (red) and metaphase cell marker pH3 (green). DNA is stained with DAPI. Dashed squares indicate magnified regions (below).

S9 Figure.

*Stra8, Sycp3* and *Dmc1* mRNA expression levels were measured by RT-qPCR in post-natal testes. Triangles represent 10 dpp values and circles represent 15 dpp values. The germ cell marker *Ddx4* was used as an endogenous reporter. Data are expressed as a percentage of the control expression level. Mean ± SEM.

S10 Figure.

High magnification of histological analysis of 10 dpp and 15 dpp testis sections stained for the meiotic marker SYCP3 and germ cell marker DDX4 as in Fig. 6A. Scale bar: 10 µm.

S1 Table: Male fertility phenotype

S2 Table: Female fertility

S3 Table: List of all primers used in the study

S4 Table: List of the antibodies used in the study
